# Photobiomodulation reduces neuropathic pain after spinal cord injury by downregulating CXCL10 expression

**DOI:** 10.1111/cns.14325

**Published:** 2023-07-20

**Authors:** Zhihao Zhang, Zhijie Zhu, Xiaoshuang Zuo, Xuankang Wang, Cheng Ju, Zhuowen Liang, Kun Li, Jiawei Zhang, Liang Luo, Yangguang Ma, Zhiwen Song, Xin Li, Penghui Li, Huilin Quan, Peipei Huang, Zhou Yao, Ning Yang, Jie Zhou, Zhenzhen Kou, Beiyu Chen, Tan Ding, Zhe Wang, Xueyu Hu

**Affiliations:** ^1^ Department of Orthopedics Xijing Hospital, Air Force Military Medical University Xi'an Shaanxi China; ^2^ 967 Hospital of People's Liberation Army Joint Logistic Support Force Dalian Liaoning China; ^3^ Department of Anatomy, Histology and Embryology, School of Basic Medicine Air Force Military Medical University Xi'an Shaanxi China

**Keywords:** CXCL10, glia cells, neuropathic pain, photobiomodulation, spinal cord injury

## Abstract

**Background:**

Many studies have recently highlighted the role of photobiomodulation (PBM) in neuropathic pain (NP) relief after spinal cord injury (SCI), suggesting that it may be an effective way to relieve NP after SCI. However, the underlying mechanisms remain unclear. This study aimed to determine the potential mechanisms of PBM in NP relief after SCI.

**Methods:**

We performed systematic observations and investigated the mechanism of PBM intervention in NP in rats after SCI. Using transcriptome sequencing, we screened CXCL10 as a possible target molecule for PBM intervention and validated the results in rat tissues using reverse transcription‐polymerase chain reaction and western blotting. Using immunofluorescence co‐labeling, astrocytes and microglia were identified as the cells responsible for CXCL10 expression. The involvement of the NF‐κB pathway in CXCL10 expression was verified using inhibitor pyrrolidine dithiocarbamate (PDTC) and agonist phorbol‐12‐myristate‐13‐acetate (PMA), which were further validated by an in vivo injection experiment.

**Results:**

Here, we demonstrated that PBM therapy led to an improvement in NP relative behaviors post‐SCI, inhibited the activation of microglia and astrocytes, and decreased the expression level of CXCL10 in glial cells, which was accompanied by mediation of the NF‐κB signaling pathway. Photobiomodulation inhibit the activation of the NF‐κB pathway and reduce downstream CXCL10 expression. The NF‐κB pathway inhibitor PDTC had the same effect as PBM on improving pain in animals with SCI, and the NF‐κB pathway promoter PMA could reverse the beneficial effect of PBM.

**Conclusions:**

Our results provide new insights into the mechanisms by which PBM alleviates NP after SCI. We demonstrated that PBM significantly inhibited the activation of microglia and astrocytes and decreased the expression level of CXCL10. These effects appear to be related to the NF‐κB signaling pathway. Taken together, our study provides evidence that PBM could be a potentially effective therapy for NP after SCI, CXCL10 and NF‐kB signaling pathways might be critical factors in pain relief mediated by PBM after SCI.

## Graphical abstract

Schematic model demonstrates the modulatory effect of photobiomodulation (PBM) on neuropathic pain (NP) in spinal cord injury (SCI) male rats and its possible molecular mechanism. The activation of the NF‐ĸB pathway after SCI increases the expression of CXCL10 in astrocytes and microglia, which would lead to the development of NP. The mechanism of PBM on the alleviation of NP after SCI might be related to its inhibition of the NF‐ĸB pathway activation in astrocytes and microglia, consequently decreases the expression of CXCL10 and alleviate NP after SCI.

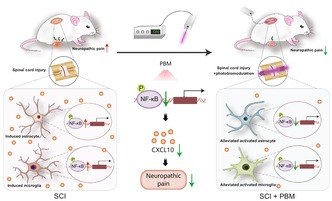

## 1 BACKGROUND

Spinal cord injury (SCI) results in disruption of afferent and efferent pathways and leads to loss of sensorimotor function below the injury site.^1^, ^2^ Neuropathic pain (NP) after SCI is a common complication^2^, ^3^, ^4^; 53% of the patients develop NP after SCI,^3^ and more than one third of them report moderate to severe pain.^4^ Accompanied by spontaneous burning, tingling, electric shock sensations, allodynia and hyperalgesia,^3^ NP after SCI could be a strong predictor of reduced quality of life.^5^

The treatment of NP following SCI is clinically challenging,^6^, ^7^ and NP is resistant to treatment with nonsteroidal anti‐inflammatory drugs and opioids.^8^ Although evidence of effective treatment by some drugs is strong, the effect size between desirable and undesirable effects is moderate to low.^8^, ^9^ Moreover, when post‐SCI pain becomes chronic, treatment becomes further difficult.^10^ Replacing or supplementing traditional pharmacological approaches with nonpharmacological interventions may be a potential choice for improving patient outcomes for NP.^10^, ^11^ However, literature on exploring the mechanisms of nonpharmacological interventions remains lacking, limiting the development of effective treatments.

Photobiomodulation (PBM) is a treatment performed using very low energy light, which is sufficient to produce stimulation, but does not damage the target system.^12^ The absorption of red/near‐infrared light enhances mitochondrial ATP production, cell signaling and growth factor synthesis, and attenuates oxidative stress and inflammation responses.^13^, ^14^ PBM is used clinically and provides therapeutical effects on diseases including Alzheimer's disease^15^ and wound care.^16^ Although no clinical studies have reported the therapeutic effects of PBM for the treatment of NP after SCI, effective relief for NP in rats after SCI has been reported in several preclinical studies.^17^, ^18^, ^19^, ^20^ An investigation into the mechanisms by which PBM alleviates the onset of NP after SCI is still needed.

Treatments reducing chemokine production or targeting chemokine receptors could be beneficial for the management of NP.^21^, ^22^ CXCL10, a classical pain‐associated chemokine, specifically binds to CXCR3 and influences the occurrence of NP.^23^, ^24^, ^25^, ^26^ Increased expression of CXCL10 occurs in nerve ligation and in the nerve chronic constriction injury model^23^, ^24^, ^25^; upregulation of CXCR3 expression is also found in spinal, trigeminal, and dorsal root ganglion neurons in animals with pain hypersensitivity.^23^, ^24^, ^26^ CXCL10 intrathecal injection or spinal cord slices perfused with CXCL10 show induced hyperalgesia and increased excitatory synaptic transmission,^26^, ^27^ while gene knockdown or inhibitor intraspinal injection markedly attenuate NP.^25^, ^26^ In humans with traumatic SCI, patients who reported pain a year after injury had higher serum levels of CXCL10 in the subacute injury period.^28^

CXCL10 transcript levels rise rapidly from 30 min to several hours after the onset of SCI,^29^, ^30^ and can be detected up to 28 days after injury.^30^ CXCL10 plays a broad role in pathophysiological changes following SCI, including recruitment of inflammatory cells, inhibition of angiogenesis, promotion of apoptosis, neuronal loss, axonal injury, and happening of NP, and impairment of motor recovery.^31^, ^32^, ^33^ CXCL10 and its receptors could be potential therapeutic target in the treatment of SCI.^34^

In the central nervous system, glial cells are involved in processes such as synaptic signaling and ion homeostasis, and provide support for neuronal metabolism in the physiological state.^35^ After injury, the interaction between central glial cells and neurons is a key factor in the pathogenesis of NP, and injury or pathological conditions that cause the onset of pain are closely related to the action of reactive microglia or astrocytes.^36^, ^37^, ^38^, ^39^ Modulation of the activation of glial cells might be helpful in alleviating NP after SCI.^40^, ^41^

Here, we investigated the potential role of PBM in pain relief after SCI and explored the potentially relevant cells and pathways contributing to it. This study suggests that PBM is a credible means of suppressing NP after SCI and tries to investigate the possible mechanism behind it.

## 2 METHODS

### 2.1 Animals

A total of 225 Sprague–Dawley rats (specific pathogen free, male, 6 weeks old) and 10 newborn rat pups were purchased from the Air Force Military Medical University Experimental Animal Center. Rats were maintained in a suitable environment and housed in individually ventilated cages free of pathogens. Animals had ad libitum access to food and ultrapure water. All animal procedures were approved by the Animal Center of the Air Force Military Medical University (approval no. 20210452, Date: March 1, 2021) and conducted in compliance with the Animal Research: Reporting of In Vivo Experiments Guideline.^42^ Rats were randomly divided into seven experimental groups, including 51 animals in the sham, SCI, and SCI + PBM groups each, and 18 animals in SCI + phorbol‐12‐myristate‐13‐acetate (PMA) group, SCI + PMA + PBM group, SCI + pyrrolidine dithiocarbamate (PDTC) group and SCI + PDTC + PBM groups each.

### 2.2 Construction of SCI model and postinjury care

#### 2.2.1 T10 clamping damage

The rats were anesthetized with sodium pentobarbital (50 mg/kg). For spinal clamp injuries, the T10 vertebra was identified according to anatomical markers, thin laminae were removed, and the spinal cord was exposed. The spinal cord was then bilaterally crushed using forceps (Fine Scientific Tools) for 40 s.^43^, ^44^, ^45^ There were no differences in the duration and operation of the injury between the experimental groups.

#### 2.2.2 PBM device placement

The front end of the laser fiber (Xi'an Laser Tech Medical Technology Co.) (see Data S1) was fixed to the T9 vertebra spinous process and its adjacent soft tissues using absorbable sutures in all rats. After connecting the rear end of the laser fiber to the laser irradiation device (Changchun Lei Shi Optoelectronics Co.) to confirm the laser could be projected directly onto the spinal cord surface, the rear end of the fiber was threaded out next to the incision and tightly sutured to the skin with absorbable sutures.^43^, ^44^

#### 2.2.3 Postinjury care

After the incision was cleaned with saline, the muscle and skin were carefully sutured layer‐by‐layer. The rats were placed on a blanket and maintained at a constant temperature until they awoke. Postoperatively, the rats were administered antibiotics via intraperitoneal injection once daily. Bladder massage was performed twice daily to prevent the development of urinary retention until spontaneous urination recovered.

### 2.3 PBM modulation

To investigate the effects of PBM treatment on NP in rats after SCI, rats were treated with PBM irradiation for 14 consecutive days, starting immediately after injury.^43^, ^44^ An 810‐nm diode laser generator (Changchun Lei Shi Optoelectronics Co.) was connected to the laser fiber fixed in the rats through a detachable device. The PBM‐treated rats received laser from the laser irradiation device with the same parameters (Table 1) each day in dark cages, as reported in previous studies.^43^, ^44^ The other rats were connected to the laser irradiation device in dark cages without irradiation.

**TABLE 1:** Parameters of laser device and photobiomodulation treatment.

Equipment parameters	Irradiation value	
	In vivo	In vitro
Center wavelength (nm)	810	810
Spectral bandwidth (nm)	<3	<3
Operation mode	Continuous mode	Continuous mode
Irradiation power (mW)	150	150
Irradiation time (min)	60	8
Irradiation energy (J)	540	72
Irradiation frequency	1/day	2/day
Treatment schedule (days)	14	0.5–1

### 2.4 PMA and PDTC administration

The NF‐κB pathway activator PMA (MCE) and NF‐κB pathway inhibitor PDTC (Selleck) were used to pharmacologically regulate the NF‐κB pathway. PMA, a type of foppo ester, was isolated from the immature fruit of Indian *Sapium indicum* and is a potent proinflammatory agent.^45^ PDTC is a thiol compound derived from dithiocarbamates that exerts anti‐inflammatory effects by inhibiting the NF‐κB pathway.^46^ For NF‐κB pathway modulating experiments, the NF‐κB pathway‐activated rats and NF‐κB pathway‐inhibited rats were intraperitoneally injected PMA (20 ng/kg)^47^ and PDTC (30 mg/kg)^48^ continuously for 7 days after injury, and other rats received vehicle injection accordingly. The body weight of the rats was measured before PMA or PDTC injection.

### 2.5 Behavior

#### 2.5.1 Open‐field movement

Two investigators who had no knowledge of the rat grouping assessed their hind limb motor function recovery in the open field using the Basso Beattie Bresnahan (BBB) scores.^49^ The BBB score is a 22‐point scale, with 0 indicating no observable movement and 21 indicating normal movement. The BBB scores of rats were evaluated preoperatively and at 1, 3, 7, 14, 21, and 28 days postinjury (dpi). At each time point, each animal was tested twice, and the BBB scores were measured independently and then averaged.

#### 2.5.2 Mechanical allodynia

Mechanical hypersensitivity in rats was assessed using an up–down paradigm measuring the threshold of paw retraction in rats^50^ and cumulative sensitivity scores (CSSs) measuring hypersensitivity over the dorsum of rats.^18^, ^19^ For Paw withdrawal threshold measurement,^50^ Von Frey filaments with hardness of 0.07, 0.16, 0.4, 0.6, 1.0, 1.4, and 2.0 g were placed on the surface of the metatarsal bone of the rat hind paw and pressed with sufficient force to bend the filaments. After 5 s of pressure, if the rats did not raise its paw, they showed a negative response, and the process was repeated with the next stiffer wire. If the rats raised their paws, a positive response was indicated, and the procedure was repeated with the next less‐stiff wire. Experimental parameters were measured six times for each rat, or stopped when four consecutive positive or negative responses occurred. The 50% mechanical retraction threshold (expressed in g) was calculated. For CSSs measurement, as previous reported,^18^, ^19^ we evaluated pain sensitivity in six regions over the dorsum of the animals to obtain the regional sensitivity scores (RSSs), which were then summed up as CSSs. First, the range of CSSs in uninjured sham animals was assessed, and a hypersensitivity threshold was defined as two standard deviations (SDs) above the mean of the sham rats. The CSSs in the sham, SCI, and SCI + PBM groups were measured to indicate mechanical allodynia.

#### 2.5.3 Acetone test

Cold nociceptive hypersensitivity in rats was assessed using the acetone evaporation test by measuring the nociceptive behavior of the hind paws following decrease in temperature due to acetone evaporation.^51^ Mice were acclimated for 1 h in an enclosure with a wire grid at the bottom. A drop of acetone was placed ventrally on each side of the hind paw of the rat using a 1‐mL syringe. The presence of nociceptive behaviors, such as lifting, shaking, licking, defense, and gnawing of the hind paws within the next 60 s was considered a positive response; otherwise, a negative response was deemed. Experiments were performed six times on each side of the hind paw, with at least 10 min between each experiment. The ratio of positive responses to the total number of experiments was calculated for both hind paws.

#### 2.5.4 Hot plate

To determine heat hyperalgesia in rats, a hot plate test was performed to measure the nociceptive response of the hind paw when exposed to excessive heat.^52^ Rats were placed on a hot plate at 55°C. When the animal showed paw retraction behavior or licked its hind paw, the test was ended, and the time was recorded.

### 2.6 Cell culture and treatment

#### 2.6.1 Culture of astrocytes, microglia, and ventral spinal cord 4.1 (VSC 4.1) motor neuron cell lines

Primary astrocytes and microglia were obtained by culturing and purification of primary mixed glial cells.^43^, ^53^ Mixed glial cells were obtained from newborn 1–2‐day rat pups and cultured in Dulbecco's Modified Eagle Medium/F‐12 (DMEM/F12; Gibco) containing 10% fetal bovine serum (Gibco) and 1% penicillin–streptomycin (Gibco) until the primary cultured mixed glial cells grew to confluence. The flasks were shaken at 200 rpm for 2 h to obtain microglia from the supernatant, and then at 200 rpm for 24 h to obtain astrocytes from the bottom of flasks. VSC4.1 motor neuron cells were grown in DMEM/F12 medium supplemented with 10% fetal bovine serum (Gibco) and 1% penicillin–streptomycin (Gibco).

#### 2.6.2 Cell treatment

Cellular inflammation induction and in vitro PBM intervention experiments were performed in astrocytes and microglia. C1q (400 ng/mL; MyBioSource), tumor necrosis factor (TNF)‐α (30 ng/mL; CST), and interleukin (IL)‐1α (3 ng/mL; Sigma‐Aldrich) were diluted in the medium of astrocytes to obtain inflammation‐induced astrocytes.^54^ Lipopolysaccharides (1 μg/mL; Sigma‐Aldrich) and interferon‐γ (20 ng/mL; Proteintech) were added to the medium of microglia to obtain inflammation‐induced microglia.^55^ PBM irradiation (see Table 1 for parameters) was performed on inflammation‐induced astrocytes and microglia at the beginning of the experiments and every 12 h afterwards.^43^, ^44^

For NF‐κB pathway modulation in astrocytes and microglia, cells that received NF‐κB pathway agonist were induced by PMA (0.6 μg/mL) for 1 h prior to the inflammation induction,^56^ and cells that received NF‐κB pathway inhibitor were modulated by PDTC (16 μg/mL) for 0.5 h prior to the inflammation induction.^57^

For VSC 4.1 motor neuron cell line induction, the effect of glutamate exposure in cultured neuron cells was induced by diluting glutamate (7 mM; Sigma‐Aldrich) in medium and incubated for 8 h.^58^, ^59^ To test whether oxygen–glucose deprivation (OGD) affects CXCL10 expression in neuronal cells, VSC 4.1 motor neuron cells were exposed to OGD for 4 h.^60^, ^61^

### 2.7 RNA sequencing (RNA‐Seq)

The total RNA from spinal cord of rats in the sham, SCI, and SCI + PBM groups were extracted with TRIzol reagent. The RIN valued of samples were tested to ensure a high quality, then the RNA samples were used to establish the RNA‐seq libraries according to the previously reported protocol^62^, ^63^, ^64^ (Data S2), The libraries were sequenced with NovaSeq 6000 (Illumina).

### 2.8 Tissue processing

The rats were anesthetized with a lethal ketamine/xylazine mixture. To obtain the tissue for immunostaining, the heart was sequentially perfused with 0.1 M phosphate‐buffered saline (PBS) and 4% paraformaldehyde. The spinal cord was removed and postfixed with 4% paraformaldehyde for 2 h. It was then transferred to 0.1 M PBS overnight, and soaked in 30% sucrose for 48 h. The spinal cord was cut into 20‐mm segments centered at the crush site. With the dorsal column on top, the tissue was embedded in a cutting medium (VWR International). After quick freezing on dry ice, the tissue was sectioned (8 μm thickness) using a Microm slicer (Leica), cut along the coronal (rostral‐caudal) axis and horizontal (dorsal‐ventral) axis, and collected on SuperFrost Plus slides (Thermo Fisher Scientific). Sections were stored at −20°C for further analysis.

To obtain the tissue for western blotting, enzyme‐linked immunosorbent assay (ELISA), and reverse transcription (RT)‐PCR, the spinal cord tissue was quickly removed and cut into 20‐mm segment centered at the crush site on an ice‐cold plate after transcardial perfusion with 0.1 M PBS solution. The tissue was carefully chopped into small pieces and homogenized in radioimmunoprecipitation assay buffer containing 0.1% protease inhibitor (Sigma‐Aldrich) and TRIzol reagent for subsequent protein and RNA acquisition respectively. Tissue samples were frozen at −80°C for further analysis.

### 2.9 RNA extraction and RT‐PCR ^65^

RNA from tissues, astrocytes, and microglia was extracted from tissue homogenates and 12‐well plates using the RNeasy Mini Kit (Qiagen). RNA concentrations of the obtained tissues and cells were determined using a spectrophotometer (Thermo Fisher Scientific). cDNA was synthesized from total RNA using Evo M‐MLV RT Premix (Accurate Biotechnology (Hunan) Co.). cDNA (10‐fold dilution) was used for RT‐PCR with primers synthesized in advance (Table S1), which was performed using a 96‐well reaction plate with an Mx3000P qPCR instrument (Agilent Technologies, Inc.) to quantify the mRNA expression levels of CXCL10, CXCR3, IL‐18, IL‐1β, TNF‐α, IL‐6, IL‐10, and Glyceraldehyde‐3‐phosphate dehydrogenase (GAPDH). RT‐PCR was performed using the following conditions: initial denaturation at 95°C for 10 min, followed by 40 cycles of denaturation at 95°C for 15 s, annealing and extension at 60°C for 60 s. The primers used in this study (Data S3) were obtained from the PubMed database and were synthesized by Shanghai Sangen Bioengineering Co. RT‐PCR data were obtained and analyzed using the relative gene expression change (2^−ΔΔCT^) method. Gene expression levels were normalized to the housekeeping gene, GAPDH. Each sample was added to three replicate wells for the assay.

### 2.10 Western blotting

Cells isolated from six‐well plates were lysed in radioimmunoprecipitation assay buffer containing protease and phosphatase inhibitors (Thermo Fisher Scientific). Protein concentrations in cells and spinal cord tissue homogenates were determined using the bicinchoninic acid.^66^ Equal amounts of protein were separated by sodium dodecyl sulfate‐polyacrylamide gel electrophoresis and transferred to nitrocellulose membranes (Sigma‐Aldrich). After blocking the membranes in 5% skim milk, they were incubated at 4°C overnight with the primary antibody including CXCL10, CXCR3, p‐P65, P65 and actin. Membranes were then incubated with secondary antibodies for 1 h at 37°C. Final staining was performed with a chemiluminescent solution. Densitometry of the immunoblots was performed using ImageJ (v1.4.67; National Institutes of Health).^67^ The following antibodies were used: rabbit anti‐CXCL10 antibody (Proteintech; 1:1000), rabbit anti‐CXCR3 antibody (Sigma‐Aldrich; 1:1000), rabbit anti‐P65 antibody (CST; 1:1000), rabbit anti‐p‐P65 antibody (CST; 1:1000), secondary anti‐rabbit antibody (InCellGene; 1:3000), and secondary anti‐mouse antibody (InCellGene; 1:3000), mouse anti‐β‐actin antibody (Proteintech; 1:3000) was used as loading control.

### 2.11 Enzyme‐linked immunosorbent assay

The expression levels of pain‐related inflammatory factors TNF‐α, IL‐1β, IL‐6, IL‐18, and IL‐10 were tested by ELISA according to a previous study.^68^ The ELISA plates were coated with the corresponding recombinant proteins in advance. Then ELISA plates were blocked using PBS containing 3% (w/v) bovine serum albumin (BSA) for 2 h, washed with PBS‐Tween 20 (0.1%) three times, and spinal cord tissue homogenate was added to the plates. After incubation for 2 h, the plates were washed and horseradish peroxidase‐conjugated secondary antibodies were added. After 1 h of incubation, the plates were washed and 3,3′,5,5′‐tetramethylbenzidine substrate was added, followed by the addition of HCl to stop the reaction. Absorbance at 405 nm was measured in a microplate reader, and the data was detected using the results from the compound holes.

### 2.12 Fluorescence immunolabeling^69^

Slides and 24‐well plates were dried at room temperature for 2 h, rinsed with 0.1 M PBS. Then the slides and 24‐well plates were blocked with PBS containing 4% BSA and 0.3% Triton X‐100 for 1 h at 37°C. The slides and 24‐well plates were incubated with the primary antibodies overnight at 4°C in a humidified chamber. After that, the slides and 24‐well plates were washed in 0.1 M PBS (3 × 4 min). Then the slides and 24‐well plates were incubated with secondary antibody diluted with PBS containing 4% BSA and 0.1% Triton X‐100. Sections were washed again with PBS (3 × 4 min), then the slides and 24‐well plates were covered with sealing solution containing 4′,6‐diamidino‐2‐phenylindole (DAPI; Beyotime). The following antibodies were used: mouse anti‐neuronal nuclear (NeuN) protein antibody (Abcam; 1:200), chicken anti‐glial fibrillary acidic protein (GFAP) antibody (Abcam; 1:400), mouse anti‐GFAP antibody (Abcam; 1:200), mouse anti‐ionizing calcium binding adapter molecule 1 (Iba1) antibody (Abcam; 1:200), rabbit anti‐CXCL10 antibody (Proteintech; 1:200), mouse anti‐CXCL10 antibody (Santa; 1:50), rabbit anti‐p‐P65 antibody (Affinity; 1:200), rabbit anti‐CXCR3 antibody (Sigma‐Aldrich; 1:200). Cy3‐conjugated affinipure goat anti‐rabbit IgG (H + L) (Proteintech; 1:200), FITC‐conjugated affinipure goat anti‐rabbit IgG (H + L) (Proteintech; 1:200), FITC affiniPure donkey anti‐chicken IgY (IgG) (H + L) (Jackson ImmunoResearch; 1:200), Alexa Fluor® 594 affiniPure goat anti‐mouse IgG (H + L) (Jackson ImmunoResearch; 1:200), and Alexa Fluor 488 affiniPure goat anti‐mouse IgG (H + L) (Jackson ImmunoResearch; 1:200).

### 2.13 Pathology

#### 2.13.1 CXCL10 and CXCR3 intensity analyses^70^

To quantify the fluorescence intensities of CXCL10 and CXCR3 in vivo, coronal and horizontal spinal cord sections of SCI rats were immune‐stained for CXCL10 and CXCR3. The sections were photographed using confocal microscope, and the images were converted to an 8‐bit format. In the coronal sections, a rectangular box centered on the crush site was drawn on the image. The box was 2 mm wide along the rostro‐caudal axis, and tall beyond the lateral edge of the spinal cord. In the horizontal sections, a 700‐μm‐wide rectangular region of interest was selected. The grayscale values of CXCL10 and CXCR3 in the selected images were measured using ImageJ software (National Institutes of Health Bethesda), and the data were exported to Microsoft Excel.

To quantify the fluorescence intensity of CXCL10 in vitro, 24‐well plates containing astrocytes and microglia were immune‐stained for CXCL10, GFAP, and Iba1. The images of 24‐well plates were photographed using microscope, and converted to an 8‐bit format. The grayscale values of CXCL10 in the image were measured in ImageJ (National Institutes of Health Bethesda), and the data were exported to Microsoft Excel.

#### 2.13.2 Percentage of CXCL10+ cells in neurons, astrocytes, and microglia^71^, ^72^

To estimate the percentage of cells expressing CXCL10 in neurons, astrocytes, and microglia in horizontal spinal cord sections after injury, the sections were immune‐stained for NeuN, GFAP, Iba1, and CXCL10. A 700‐μm‐wide region of interest was selected at the same anatomical location on horizontal sections at the edge of the lesion (delineated by dense astrocytes) in different rat samples. The total number of neurons, astrocytes, or microglia cells, and the number of CXCL10+ cells were counted manually. The number of CXCL10+ cells for each cell type was divided by the total number of that cell type to obtain the percentage of CXCL10+ cells in neurons, astrocytes, or microglia.

#### 2.13.3 Percentage of p‐P65+ cells^73^

Immunofluorescence staining for DAPI and p‐P65 was performed on horizontal slides followed by imaging. The total number of DAPI‐labeled nuclei and the number of p‐P65 and DAPI double‐labeled nuclei were counted manually, and the percentage of p‐P65 co‐labeled nuclei was calculated by dividing the number of double‐labeled nuclei by the total number of nuclei.

### 2.14 Statistics

GraphPad Prism (version 9.0.0; GraphPad Software, www.graphpad.com) was used for data visualization and statistical analysis. The Shapiro–Wilk test was used to test the normality of the data. One‐way analysis of variance with Bonferroni post‐hoc test was used to compare one variable in three or more groups. Two‐way analysis of variance with Bonferroni post‐hoc test was used to compare two variables in two or more groups. All data are shown as the mean ± SD. Statistical significance was set at *p* < 0.05. Images were obtained using the Adobe Illustrator (version 25.2.3, Adobe, www.adobe.com).

## 3 RESULTS

### 3.1 PBM alleviates NP‐related behaviors, promotes motor function recovery, and reduces levels of NP‐related molecules after SCI in animal model

**FIGURE 1:**
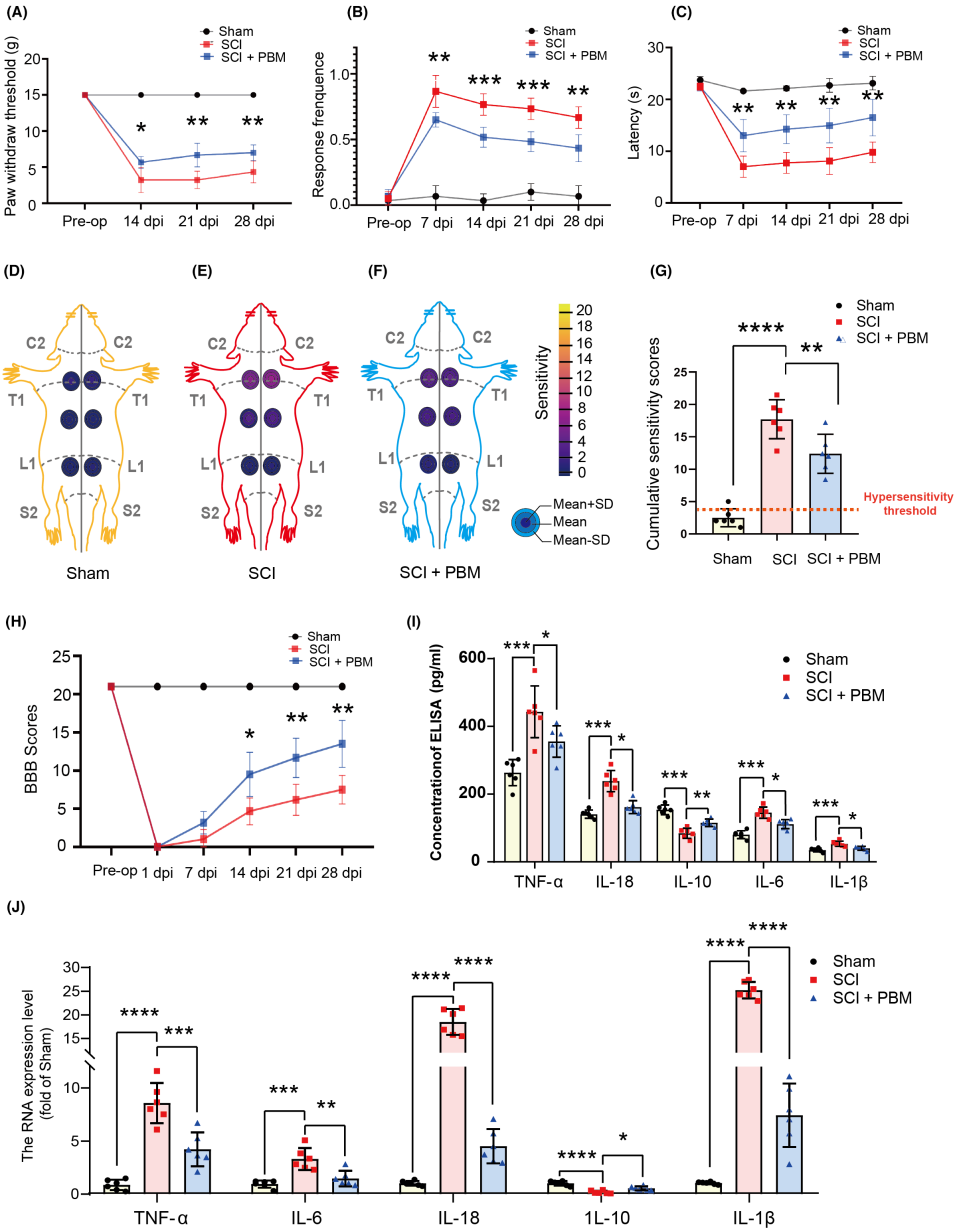
Effect of PBM irradiation on nociceptive hypersensitivity, motor function recovery and expression level of inflammatory factors. The time course of mechanical allodynia (A), cold allodynia (B) and heat hyperalgesia (C) of rats with SCI. The RSSs for the sham group (*n* = 6) (D), SCI group (*n* = 6) (E), and SCI + PBM group (*n* = 6) (F) are shown in the six regions (left and right side above‐level of injury, at the level of injury, and below‐level of injury) on rat dorsal schematic, the gray lines were used to write C2, T1, L1, S2, and midline on that. The number of mean ± SD is corresponding to the colors of the concentric circles on the dorsal schematic, and a color scale depicting the number is shown in the legend. CSSs in the sham animals (*n* = 6) and SCI animals with (*n* = 6) or without (*n* = 6) PBM irradiation are accumulated by RSSs (G); red‐dashed line indicates hypersensitivity threshold (3.76), which is taken from the control group. The BBB score was used to evaluate the recovery of motor function in the sham group (*n* = 6), SCI group (*n* = 6) and SCI + PBM group (*n* = 6) (H). Fold changes in the expression (I) and transcription (J) levels of the representative inflammatory factors associated with the occurrence of pain (TNF‐α, IL‐6, IL‐18, IL‐10, and IL‐1β) in the sham group (*n* = 6), SCI group (*n* = 6) and SCI + PBM group (*n* = 6) at 7 dpi. Data is expressed as mean ± SD, *p < 0.05, **p < 0.01, ***p < 0.001, ****p < 0.0001. BBB, Basso, Beattie, Bresnahan; CSS, cumulative sensitivity score; dpi, days postinjury; PBM, photobiomodulation; RSS, regional sensitivity score; SCI, spinal cord injury; SD, standard deviation.

In the rat model experiment, PBM was administered for 14 consecutive days after SCI to observe its effect on NP relief at 7, 14, 21, and 28 dpi. PBM significantly alleviated mechanical allodynia, cold allodynia, and heat hyperalgesia in SCI rats (Figure 1A–C; Table 2). CSSs scores were identified at 7 dpi in rats of the sham group, SCI group, and SCI + PBM group (Figure 1D–F); most animals in the sham group had category 1 and 2 responses with a mean ± SD CSS of 2.67 ± 0.548, and the hypersensitivity threshold was 3.762 (mean + 2SD). Further, CSSs were quantified in SCI and SCI + PBM groups (Figure 1G; Table 2). The BBB score was used to evaluate the effect of PBM on functional recovery after SCI,^49^ and the BBB scores in the SCI group markedly improved at 14, 21, 28 dpi (Figure 1H).

**TABLE 2:** PBM significantly alleviated mechanical allodynia, cold allodynia, and heat hyperalgesia in SCI rats.

NP‐related behaviors	SCI vs. SCI + PBM								*F* value	*p* Value
	7 dpi		14 dpi		21 dpi		28 dpi			
Mechanical allodynia										
Paw withdraw threshold			3.233 ± 1.745 vs. 5.667 ± 0.817	*p* = 0.0172	3.233 ± 1.203 vs. 6.667 ± 1.633	*p* = 0.0024	4.333 ± 1.506 vs. 7.000 ± 1.095	*p* = 0.0065	*F* (6, 40) = 57.69	<0.0001
Cumulative sensitivity scores									*F* (2, 15) = 53.48	<0.0001
Cold allodynia	0.867 ± 0.121 vs. 0.650 ± 0.0548	*p* = 0.0053	0.7667 ± 0.08165 vs. 0.5167 ± 0.07528	*p* = 0.0003	0.7333 ± 0.08165 vs. 0.4833 ± 0.07528	*p* = 0.0003	0.6667 ± 0.08165 vs. 0.4333 ± 0.1033	*p* = 0.0017	*F* (8, 60) = 27.80	<0.0001
Heat hyperalgesia	7.033 ± 2.052 vs. 13.03 ± 3.108	*p* = 0.0037	7.767 ± 2.028 vs. 14.27 ± 2.765	*p* = 0.0012	8.117 ± 2.579 vs. 14.95 ± 3.330	*p* = 0.0030	9.800 ± 1.971 vs. 16.500 ± 3.534	*p* = 0.0038	*F* (8, 60) = 26.26	<0.0001

Abbreviations: dpi, days postinjury; NP, neuropathic pain; PBM, photobiomodulation; SCI, spinal cord injury.

To further investigate the role of inflammatory responses in the reduction of NP by PBM, the transcription and expression of inflammatory factors were examined. ELISA results (Figure 1I) showed that the expression levels of pain‐related inflammatory factors were significantly increased at 7 dpi, and PBM intervention inhibited the increase in these molecules to varying degrees [TNF‐α, *F* (2, 15) = 15.36, *p* = 0.0002; IL‐1β, *F* (2, 15) = 13.04, *p* = 0.0005; IL‐18, *F* (2, 15) = 32.06, *p* < 0.0001; IL‐6, *F* (2, 15) = 32.54, *p* < 0.0001]. In addition, the expression level of IL‐10 was significantly increased after PBM treatment in SCI rats [*F* (2, 15) = 39.73, *p* < 0.0001]. RT‐PCR results (Figure 1J) also showed that SCI led to increased TNF‐α, IL‐1β, IL‐18, and IL‐6 transcription levels in rat tissues, whereas PBM inhibited this increase [TNF‐α, *F* (2, 15) = 42.26, *p* < 0.0001; IL‐1β, *F* (2, 15) = 16.08, *p* = 0.0002; IL‐18, *F* (2, 15) = 152.1, *p* < 0.0001; IL‐6, *F* (2, 15) = 16.08, *p* = 0.0002], and the IL‐10 expression levels were reversed after PBM treatment [*F* (2, 15) = 30.42, *p* < 0.0001].

### 3.2 CXCL10 upregulation was suppressed by PBM after SCI

**FIGURE 2:**
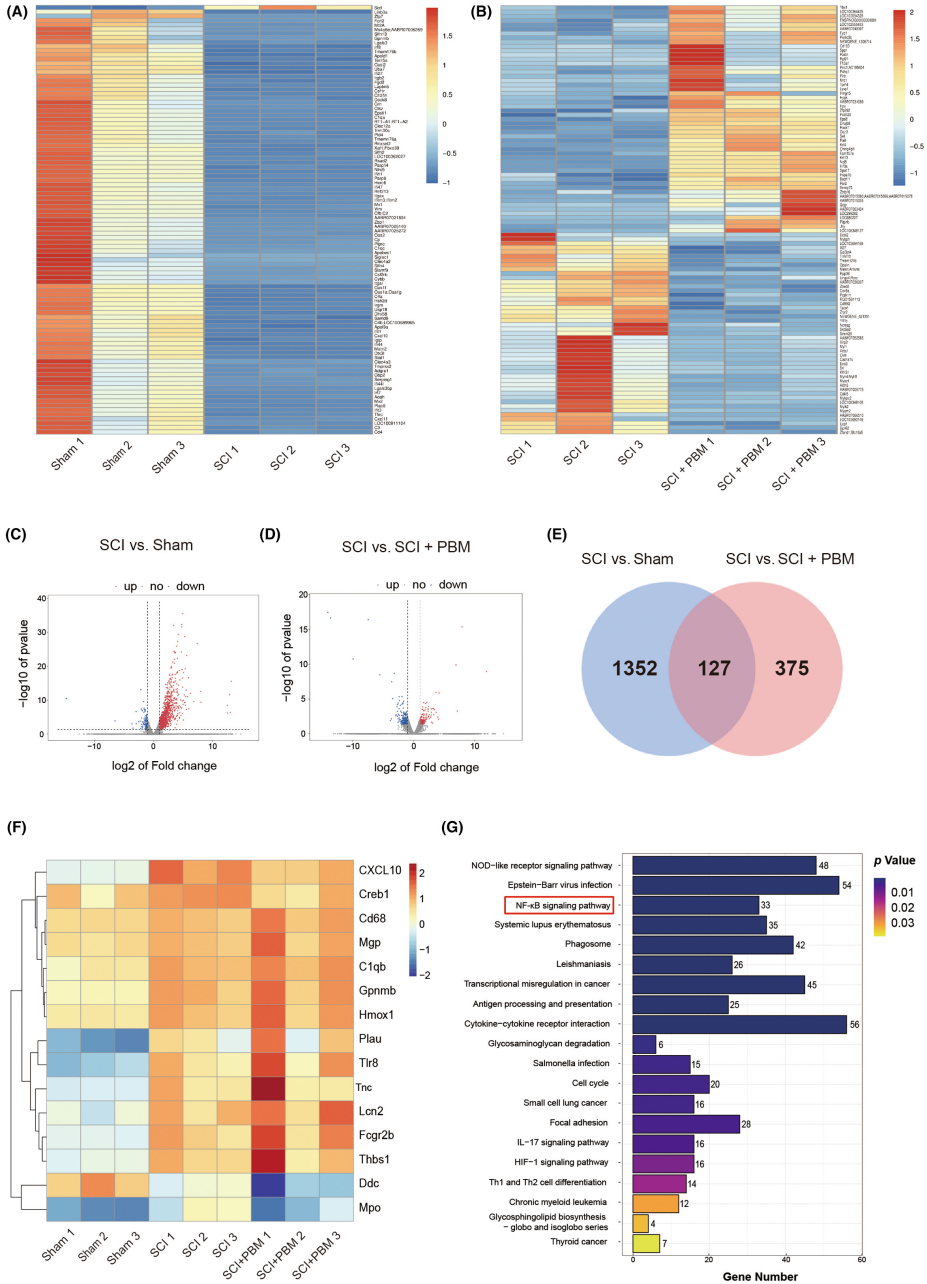
RNA‐seq analysis of the effect of PBM on SCI animals at 7 dpi. Gene transcriptional differences in the sham group and SCI group is shown in the heat map (A), with 1242 genes significantly upregulated and 110 genes significantly downregulated (C). Gene transcriptional differences in the SCI and SCI + PBM group is shown in the heat map (B), with 175 genes significantly upregulated and 200 genes significantly downregulated (D). Total number of genes significantly altered after SCI is 1352, and the number of genes could be modified by PBM intervention after SCI is 375. The transcript level of 127 genes significantly altered after SCI and could be modified by PBM intervention (E). Heatmap indicates CXCL10 is the top gene highly upregulated after SCI and marked decreased after PBM irradiation (F). KEGG pathway analysis between the SCI and the sham groups showed that the NF‐κB signaling pathway was dramatically activated after SCI (G). dpi, days postinjury; KEGG, Kyoto Encyclopedia of Genes and Genomes; PBM, photobiomodulation; RNA‐seq, RNA sequencing; SCI, spinal cord injury.

**FIGURE 3:**
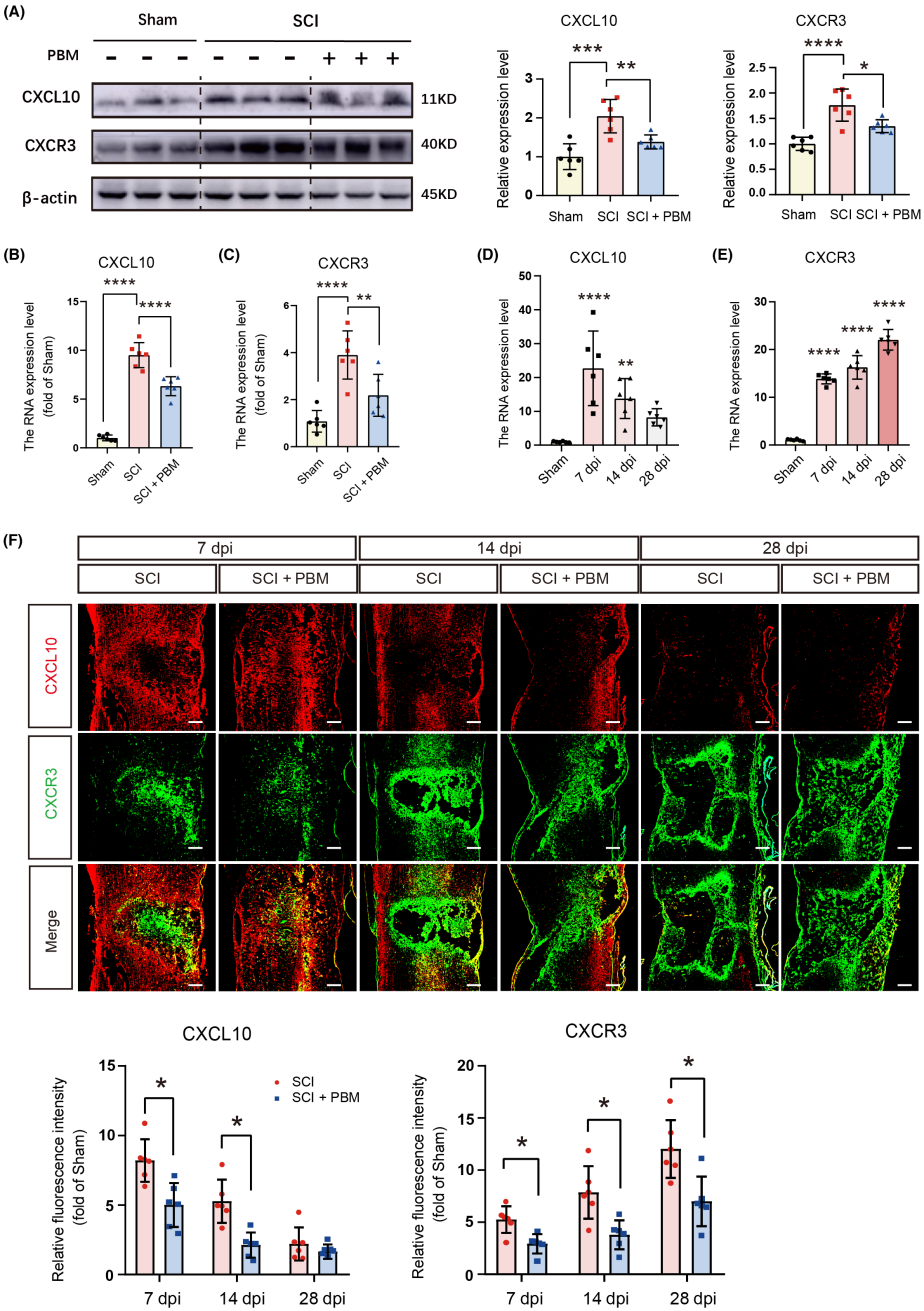
Spatial and temporal distribution of CXCL10 in rats with SCI and PBM intervention. Representative western blotting results for expression level of CXCL10 and CXCR3 in the sham group (*n* = 6), SCI group (*n* = 6), and SCI + PBM group (*n* = 6) at 7 dpi (A). Transcription levels of CXCL10 (B) and CXCR3 (C) at 7 dpi in the sham group (*n* = 6), SCI group (*n* = 6), and SCI + PBM group (*n* = 6). Quantification of relative expression level of CXCL10 (D) and CXCR3 (E) in the sham group (*n* = 6) and SCI group at 7, 14 and 28 dpi (*n* = 6 for each time point). Representative image of immunofluorescence stain for CXCL10, CXCR3 in the sham group (*n* = 6 for each time point), SCI group (*n* = 6 for each time point), and SCI + PBM group (*n* = 6 for each time point) at 7, 14 and 28 dpi. Scale bar for all pictures: 200 μm (F). Data is expressed as mean ± SD, *p < 0.05, **p < 0.01, ***p < 0.001, ****p < 0.0001. dpi, days postinjury; PBM, photobiomodulation; SCI, spinal cord injury.

To extensively explore the pathogenesis of PBM in alleviating NP after SCI, we examined transcriptional changes in spinal cord in the sham, SCI, and SCI + PBM groups by RNA‐Seq. As the repressive effect of PBM on NP could be observed from 7 dpi, RNA‐Seq was performed at 7 dpi, and showed that 1242 genes were upregulated, and 110 genes were downregulated in the SCI group compared with the sham group (Figure 2A,C), 175 genes were upregulated, and 200 genes were downregulated in the SCI + PBM group compared with the SCI group (Figure 2B,D). Among the 1352 SCI‐regulated genes whose expression levels were altered after SCI, and 375 PBM‐regulated genes whose expression levels were changed after PBM irradiation, we selected 127 target genes contained in both groups (Figure 2E). In 127 target genes, we identified genes associated with the occurrence of pain and selected the top gene CXCL10 in them as the potential target gene associated with the occurrence of NP and PBM regulation after SCI. Notably, we observed that the chemokine CXCL10 was highly expressed after SCI and was significantly downregulated following PBM treatment (Figure 2F). Kyoto Encyclopedia of Genes and Genomes (KEGG) analysis showed that the NF‐κB signaling pathway, a known pain‐related pathway,^74^, ^75^, ^76^, ^77^ was markedly upregulated in the SCI group compared with the sham group (Figure 2G). We further confirmed the RNA‐Seq results with western blotting [Figure 3A, *F* (2, 15) = 15.38, *p* = 0.0002, CXCL10; *F* (2, 15) = 19.79, *p* < 0.0001, CXCR3] and RT‐PCR [Figure 3B,C, *F* (2, 15) = 124, *p* < 0.0001, CXCL10; *F* (2, 15) = 17.73, *p* = 0.0001, CXCR3] using spinal cord tissue of animals in the 7‐day SCI group and the 7‐day SCI + PBM group.

### 3.3 Spatiotemporal expression of CXCL10 in SCI animals

To further explore the dynamics of CXCL10 expression in the spinal cord after SCI, we assessed the expression of CXCL10 and CXCR3 at different time points postinjury (Figure 3D,E). RT‐PCR results showed that CXCL10 expression in the SCI group increased significantly at 7 dpi and then decreased gradually with time [*F* (3, 20) = 12.42, *p* < 0.0001], while the expression level of CXCR3 gradually increased over time [*F* (3, 20) = 158.5, *p* < 0.0001]. The results of immunofluorescence staining performed at different times after injury were consistent with this finding [Figure 3F, *F* (4, 40) = 2.805, *p* = 0.0383, CXCL10; *F* (4, 40) = 3.319, *p* = 0.0194, CXCR3]. The expression level of CXCL10 was markedly downregulated at 7 and 14 dpi, and the expression level of CXCR3 was significantly reduced at 7, 14, and 28 dpi in the SCI + PBM group compared with the SCI group.

**FIGURE 4:**
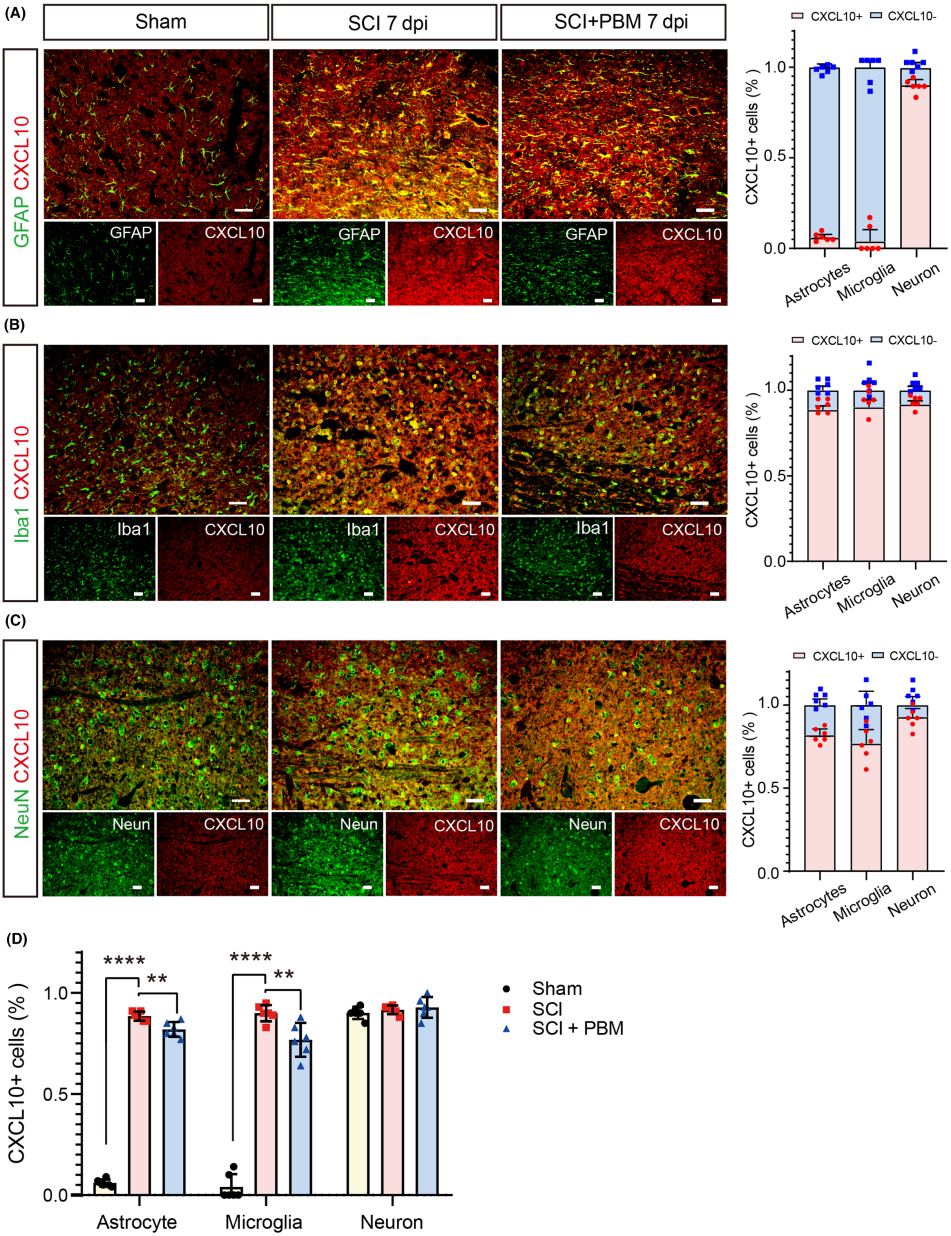
Expression levels of CXCL10 in astrocytes, microglia, and neurons in the sham, SCI, and SCI + PBM groups rats at 7 dpi. Co‐stain of CXCL10 and astroglia marker GFAP (A), microglial marker Iba1 (B), and neuronal marker NeuN (C) in representative views in the sham group (*n* = 6), SCI group (*n* = 6), and SCI + PBM group (*n* = 6) at 7 dpi. Scale bar for all pictures: 50 μm. Quantification of the proportion of CXCL10+ cells in astrocytes, microglia, and neurons in the sham group (*n* = 6), SCI group (*n* = 6), and SCI + PBM group (*n* = 6) at 7 dpi (D). Data is expressed as mean ± SD, **p < 0.01, ****p < 0.0001. dpi, days postinjury; PBM, photobiomodulation; SCI, spinal cord injury; SD, standard deviation.

To further detect the expression and distribution of CXCL10 in spinal cord tissue in the sham, SCI, and SCI + PBM groups, we used immunostaining to co‐stain CXCL10 with GFAP (Figure 4A), Iba‐1 (Figure 4B), and NeuN (Figure 4C). In the sham group, CXCL10+ cells had the highest proportion of cells stained with NeuN, for Iba‐1+ and GFAP+ cells, the CXCL10+ proportion was relatively low. In the SCI group, the proportion of CXCL10+ cells was high in all three cell types. PBM intervention significantly downregulated the proportion of CXCL10+ cells in astrocytes and microglia but not in neurons (Figure 4D; Table 3). These results indicate that CXCL10 is constitutively expressed in neurons, while in astrocytes and microglia, the expression level is upregulated after SCI and reduced after PBM irradiation.

**TABLE 3:** Expression and distribution of CXCL10 in astrocytes, microglia, and neurons.

	Percentage of CXCL10+ cells			*F* value	*p* value
	Sham group	SCI group	SCI + PBM group		
Astrocytes	0.06 ± 0.018	0.885 ± 0.02258	0.82 ± 0.03688	*F* (2, 15) = 1729	<0.0001
Microglia	0.04 ± 0.063	0.9 ± 0.04099	0.7683 ± 0.084	*F* (2, 15) = 303.2	<0.0001
Neurons	0.9017 ± 0.030	0.9167 ± 0.0216	0.9283 ± 0.05115	*F* (2, 15) = 0.8082	0.4641

Abbreviations: PBM, photobiomodulation; SCI, spinal cord injury.

### 3.4 Expression of CXCL10 was upregulated in astrocytes and microglia with the interaction of inflammatory induction and reduced by PBM irradiation

**FIGURE 5:**
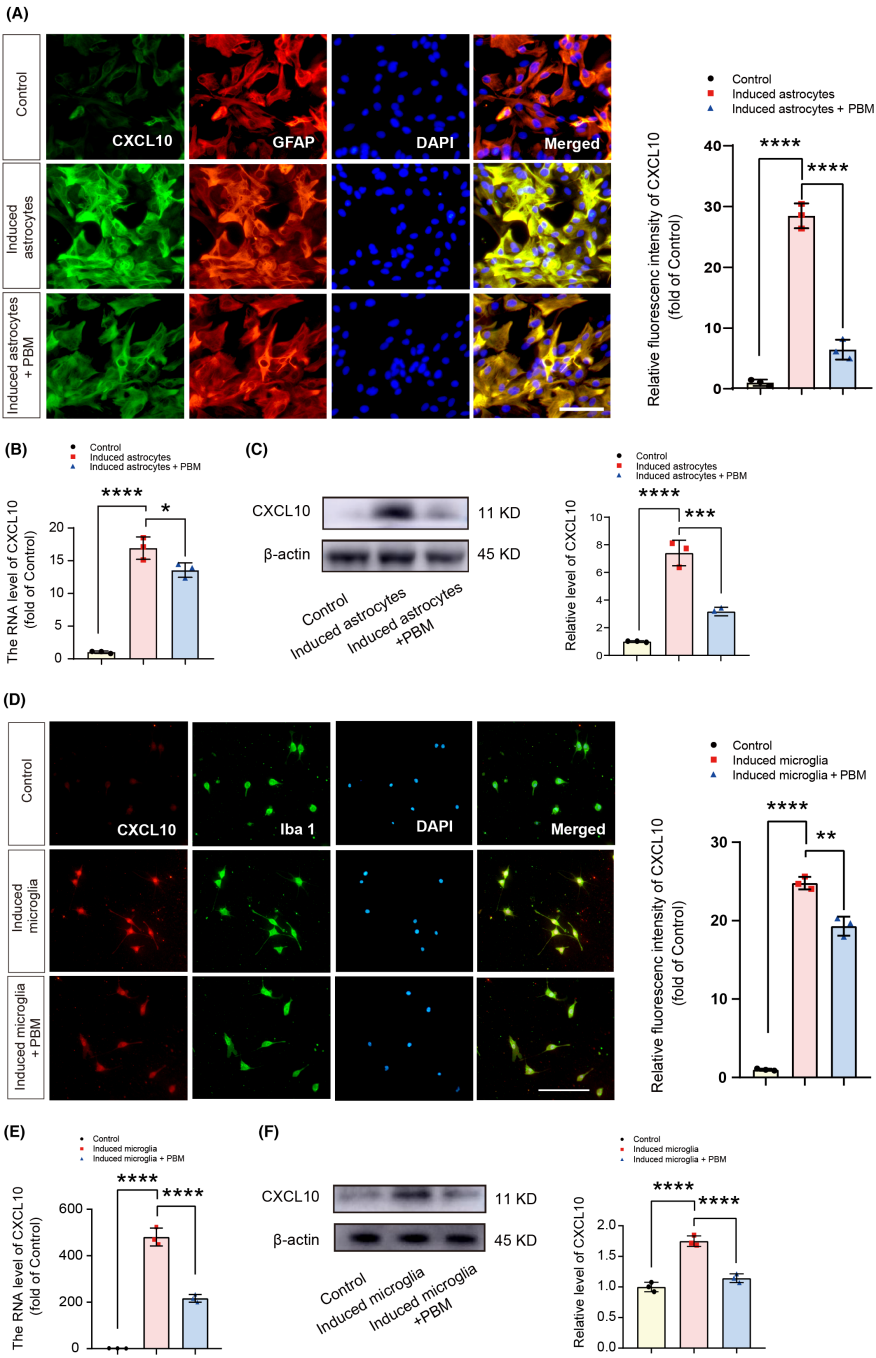
PBM inhibited the CXCL10 expression in microglia and astrocytes in vitro. Representative views of immunofluorescence staining for CXCL10 and GFAP in the control, induced astrocytes, and induced astrocytes + PBM groups and the quantification of the fluorescence intensity of CXCL10. Scale bar for all pictures: 200 μm (A). Transcript levels of CXCL10 in the control, induced astrocytes, and induced astrocytes + PBM groups (B). Representative western blotting and quantification of the relative expression level of CXCL10 in the control, induced astrocytes, and induced astrocytes + PBM groups (C). Representative views of immunofluorescence staining for CXCL10 and Iba1 in the control, induced microglia, and induced microglia + PBM groups and the quantification of the fluorescence intensity of CXCL10. Scale bar for all pictures: 200 μm (D). Transcription levels of CXCL10 in the control, induced microglia and induced microglia + PBM groups (E). Representative western blotting and quantification of the relative expression level of CXCL10 in the control, induced microglia, and induced microglia + PBM groups (F). Data is expressed as mean ± SD, *p < 0.05, **p < 0.01, ***p < 0.001, ****p < 0.0001. PBM, photobiomodulation; SCI, spinal cord injury; SD, standard deviation.

To further verify the inhibitory effect of PBM on CXCL10 expression in astrocytes and microglia, we activated microglia and astrocytes in vitro by mimicking neuroinflammatory condition, then the induced cells were irradiated with PBM. We first cultured microglia and astrocytes and confirmed the purity of the cells (see Data S4). Next, we inflammatory‐induced microglia using lipopolysaccharides and interferon‐γ, and induced astrocytes with C1q, TNF‐α, and IL‐1α. PBM irradiation was then performed to verify its effect on CXCL10 expression in inflammatory‐induced microglia and astrocytes. RT‐PCR, immunofluorescence, and western blotting results showed that CXCL10 expression was significantly upregulated in inflammatory‐induced astrocytes, and PBM irradiation reduced its levels [Figure 5A–C, *F* (2, 6) = 269.9, *p* < 0.0001, immunofluorescence; *F* (2, 6) = 152.8, *p* < 0.0001, RT‐PCR; *F* (2, 6) = 101.5, *p* < 0.0001, western blotting]. Similarly, PBM reversed elevated CXCL10 expression in inflammatory‐induced microglia [Figure 5D–F, *F* (2, 6) = 647.2, *p* < 0.0001, immunofluorescence; *F* (2, 6) = 295.8, *p* < 0.0001, RT‐PCR; *F* (2, 6) = 77.55, *p* < 0.0001, western blotting]. VSC 4.1 motor neuron cell line was induced with the glutamate excitotoxicity model and OGD model in vitro, and no significant difference was observed in CXCL10 expression in glutamate induced groups and OGD induced groups compared with the control groups (Data S5).

### 3.5 NF‐ĸB pathway activation was affected by PBM in the induced astrocytes and microglia in vitro

**FIGURE 6:**
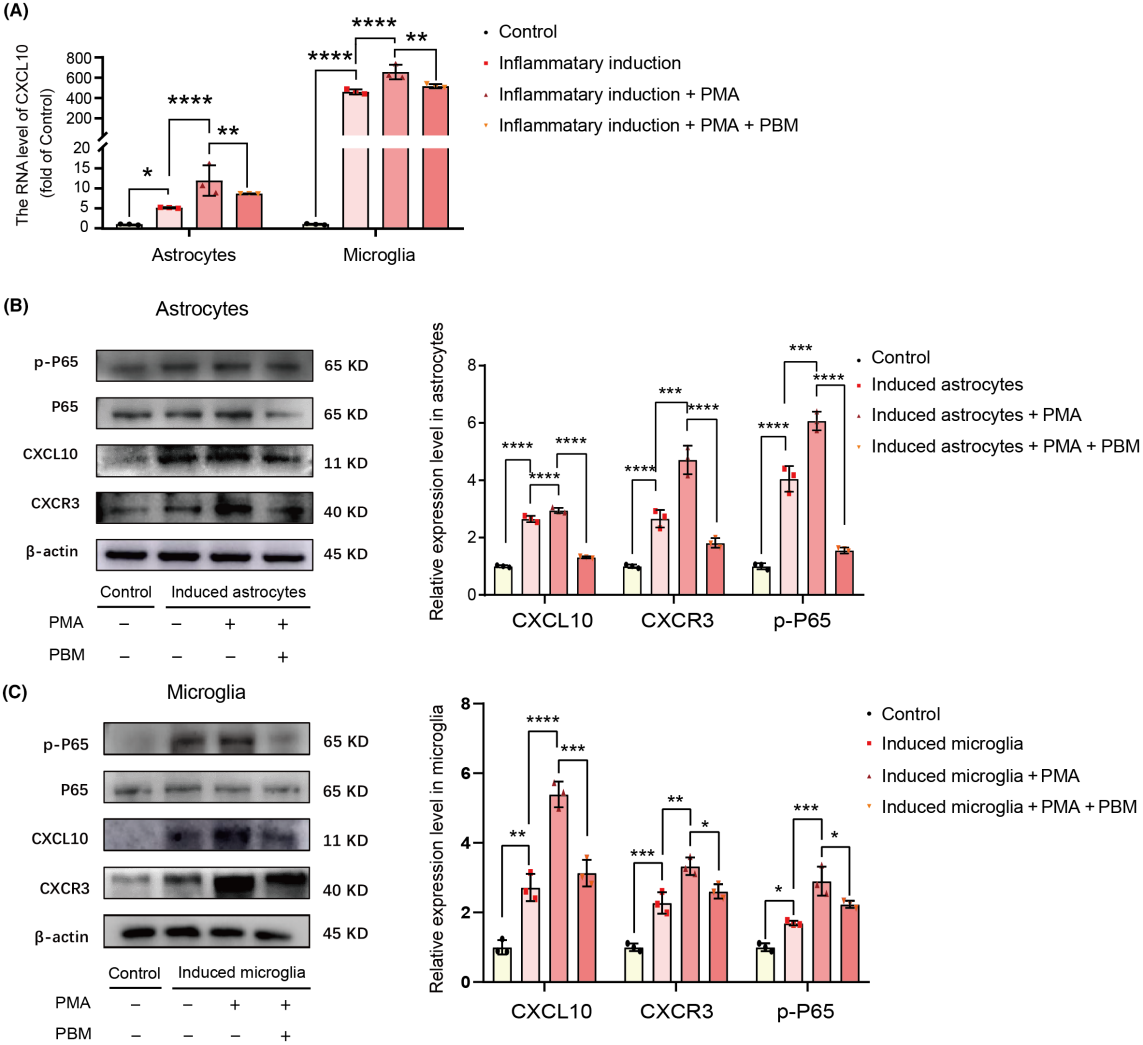
PBM suppressed the upregulating effect of PMA in the expression of CXCL10 in vitro. PMA significantly upregulated the transcription level of CXCL10 and PBM significantly suppressed this effect (A). The representative western blotting and quantification of the relative expression level of p‐P65, CXCL10, and CXCR3 in the control, induced astrocytes/microglia, induced astrocytes/microglia + PMA and induced astrocytes/microglia + PMA + PBM groups indicated the expression level of p‐P65, CXCL10 and CXCR3 in induced astrocytes/microglia + PMA group was markedly downregulated by PBM (B, C). Data is expressed as mean ± SD, *p < 0.05, **p < 0.01, ***p < 0.001, ****p < 0.0001. PBM, photobiomodulation; PMA, phorbol 12‐myristate 13‐acetate; SD, standard deviation.

To better understand the signaling pathway of PBM intervention on CXCL10 expression in SCI, we explored the pathway enrichment in KEGG. KEGG results showed that the NF‐ĸB pathway changed significantly at 7 dpi (Figure 2G). Previous studies have shown that the NF‐ĸB pathway is a classical signaling pathway involved in CXCL10 synthesis in different central nervous system diseases and injury models.^78^, ^79^, ^80^ To explore the effects of PBM on the regulation of NF‐ĸB pathway, we designed an in vitro validation experiment. PMA was added to the media of astrocytes and microglia to activate the NF‐ĸB pathway before inflammatory induction. The transcription level of CXCL10 in the inflammatory‐induced group was further upregulated after the application of PMA and downregulated after the application of PBM [Figure 6A *F* (3, 8) = 39.5, *p* < 0.0001, astrocytes; *F* (3, 8) = 153.1, *p* < 0.0001, microglia], In addition, accompanied with the increased expression level of p‐P65 in astrocytes and microglia, the expression level of CXCL10 and CXCR3 also increased, and PBM intervention reversed this process [Figure 6B,C astrocytes: *F* (3, 8) = 201.4, *p* < 0.0001, p‐P65/P65; *F* (3, 8) = 475.1, *p* < 0.0001, CXCL10; *F* (3, 8) = 81.71, *p* < 0.0001, CXCR3; microglia: *F* (3, 8) = 38.57, *p* < 0.0001, p‐P65/P65; *F* (3, 8) = 81.62, *p* < 0.0001, CXCL10; *F* (3, 8) = 53.21, *p* < 0.0001, CXCR3].

**FIGURE 7:**
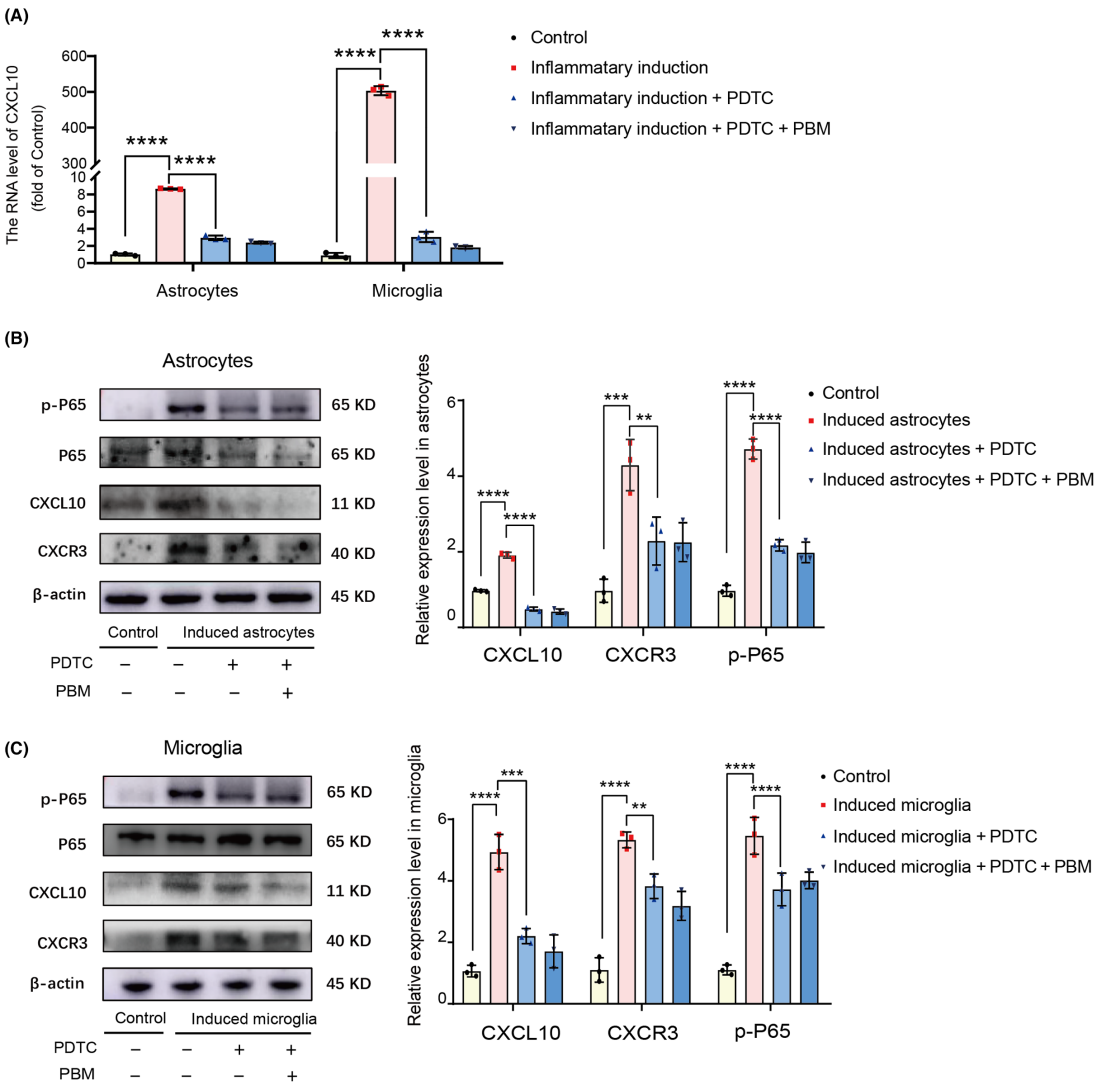
Both PDTC and PBM had inhibitory effect in the expression of CXCL10 in vitro. PBM had the same effect as PDTC on downregulating the transcription level of CXCL10 (A). The representative western blotting and quantification of the relative expression level of p‐P65, CXCL10 and CXCR3 in the control, induced astrocytes/microglia, induced astrocytes/microglia + PDTC and induced astrocytes/microglia + PDTC + PBM groups showed that the expression level of p‐P65, CXCL10 and CXCR3 in induced astrocytes/microglia group could be markedly downregulated by both PBM and PDTC (B, C). Data is expressed as mean ± SD, **p < 0.01, ***p < 0.001, ****p < 0.0001. PBM, photobiomodulation; PDTC, ammonium pyrrolidine dithiocarbamate; SCI, spinal cord injury; SD, standard deviation.

Next, we used the pathway inhibitor PDTC in vitro to block this pathway in astrocytes and microglia before inflammatory induction. PDTC is a promising molecule that has been shown to significantly reduce activation of the NF‐ĸB pathway.^81^ The transcription level of CXCL10 in the inflammatory‐induced group was downregulated in the groups exposed to PDTC or PBM irradiation [Figure 7A *F* (3, 8) = 1235, *p* < 0.0001 astrocytes; *F* (3, 8) = 970.2, *p* < 0.0001 microglia]. The expression level of p‐P65 was decreased in the inflammation‐induced + PDTC group compared with that in the inflammation‐induced group, accompanied with decrease of the expression levels of CXCL10 and CXCR3. The effects of PBM were comparable to those of PDTC [Figure 6B,C astrocytes: *F* (3, 8) = 164.7, *p* < 0.0001, p‐P65/P65; *F* (3, 8) = 402.4, *p* < 0.0001, CXCL10, *F* (3, 8) = 18.58, *p* = 0.0006, CXCR3; microglia: *F* (3, 8) = 53.50, *p* < 0.0001, p‐P65/P65; *F* (3, 8) = 49.02, *p* < 0.0001, CXCL10; *F* (3, 8) = 60.77, *p* < 0.0001, CXCR3].

### 3.6 Effects of PBM on NF‐ĸB signaling pathway and NP‐related behaviors in animals with SCI

**FIGURE 8:**
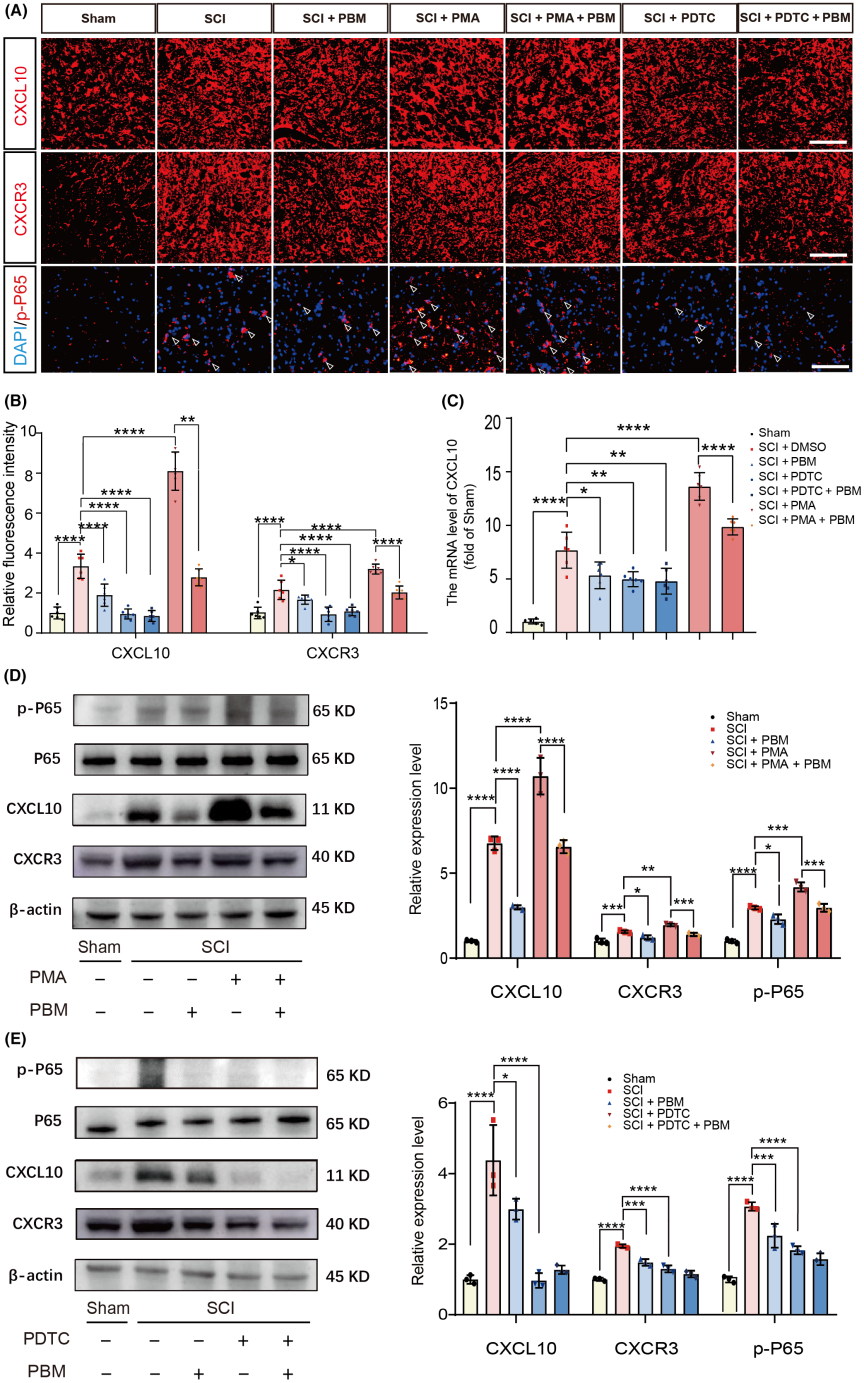
PBM reduces the expression of CXCL10 via NF‐ĸB signaling pathway in vivo. Representative views of immunofluorescence staining of CXCL10, CXCR3, p‐P65 in the sham, SCI, SCI + PBM, SCI + PMA, SCI + PMA + PBM, SCI + PDTC, and SCI + PDTC + PBM groups at 7 dpi (*n* = 6 for each group). Scale bar for all pictures: 200 μm (A, B). PBM significantly suppressed the transcription levels of CXCL10 in SCI and SCI + PMA groups (*n* = 6 for each group), and had the same effect with PDTC (C). Representative western blotting results and quantification of the relative expression level of p‐P65, CXCL10 and CXCR3 in the sham, SCI, SCI + PBM, SCI + PMA, SCI + PMA + PBM, SCI + PDTC, and SCI + PDTC + PBM groups (*n* = 6 for each group) at 7 dpi were demonstrated (D, E). Data is expressed as mean ± SD, *p < 0.05, **p < 0.01, ***p < 0.001, ****p < 0.0001. dpi, days postinjury; PBM, photobiomodulation; PDTC, ammonium pyrrolidine dithiocarbamate; PMA, phorbol 12‐myristate 13‐acetate; SCI, spinal cord injury; SD, standard deviation.

**FIGURE 9:**
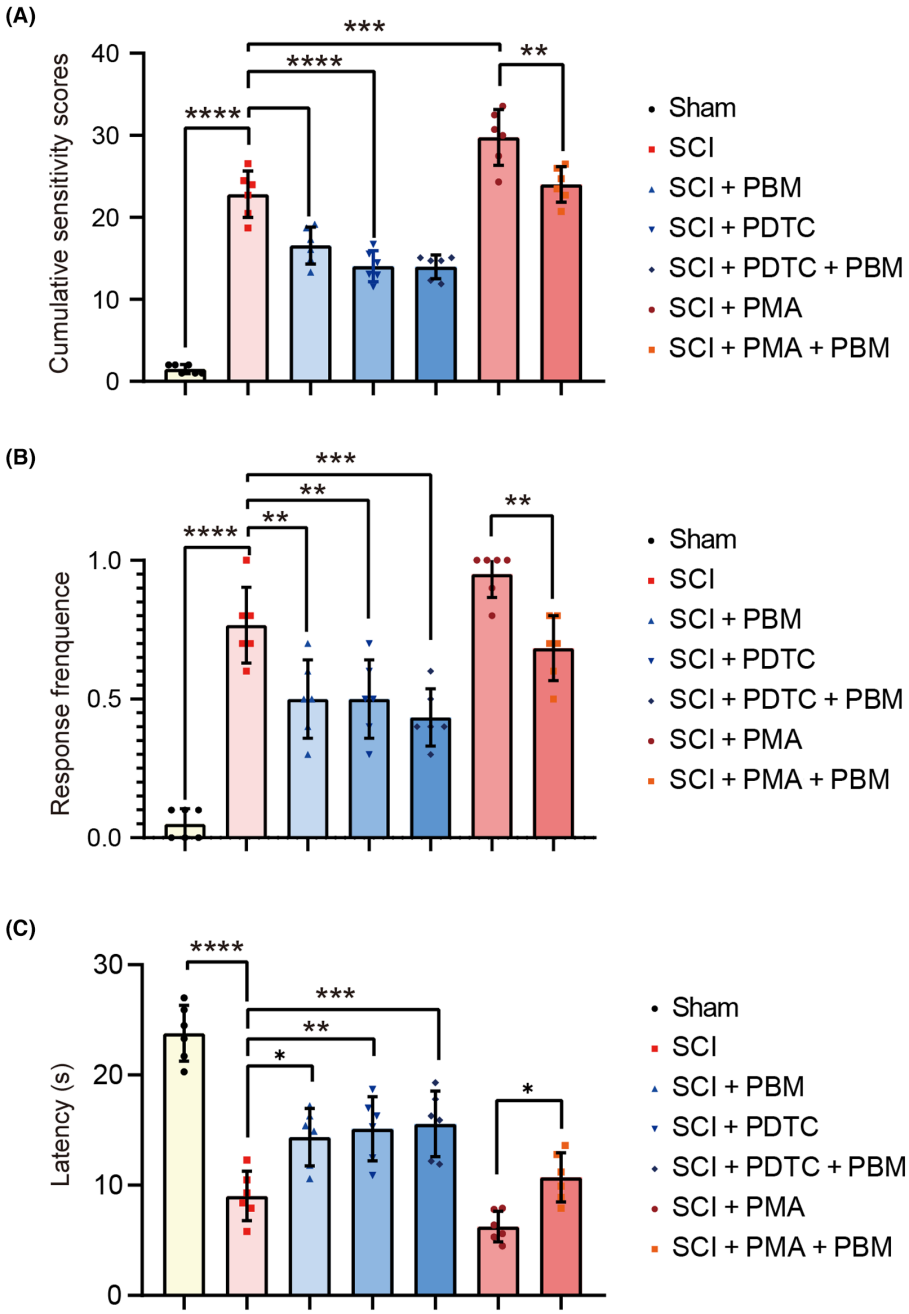
PBM alleviates NP‐related behaviors via NF‐ĸB signaling pathway in rats. Behavior scores of rats in the sham, SCI, SCI + PBM, SCI + PMA, SCI + PMA + PBM, SCI + PDTC, and SCI + PDTC + PBM groups (*n* = 6 for each group) at 7 dpi for mechanical allodynia (A), heat hyperalgesia (B), and cold allodynia (C) were investigated. Data is expressed as mean ± SD, *p < 0.05, **p < 0.01, ***p < 0.001, ****p < 0.0001. dpi, days postinjury; NP, neuropathic pain; PBM, photobiomodulation; PDTC, ammonium pyrrolidine dithiocarbamate; PMA, phorbol 12‐myristate 13‐acetate; SCI, spinal cord injury; SD, standard deviation.

We verified the effect of PBM on the NF‐ĸB pathway in vivo at 7 dpi. Immunofluorescence staining revealed that PMA increased the expression levels of CXCL10 and CXCR3 and the number of nuclei co‐stained with p‐P65 in postinjury tissues, which was reversed by PBM irradiation. PDTC suppressed the expression of CXCL10 and CXCR3 and decreased the number of nuclei co‐stained with p‐P65 similar with the effect of PBM irradiation [Figure 8A,B, *F* (6, 35) = 138.2, *p* < 0.0001, CXCL10; *F* (6, 35) = 39.94, *p* < 0.0001, CXCR3]. Then the effects of PMA, PDTC, and PBM on activation of the NF‐ĸB pathway and CXCL10 expression were quantified. The transcription level of CXCL10 in group exposure with PMA was further upregulated compared with that in SCI group, and decreased after PBM application. PBM intervention had the same effect of PDTC [Figure 8C, *F* (7, 16) = 49.82, *p* < 0.0001]. Then we compared the expression of p‐P65/P65, CXCL10, and CXCR3 at 7 dpi with western blotting. Compared to the SCI group, rats in the SCI + PMA group showed higher activation level of the NF‐ĸB pathway and a marked increase of CXCL10 expression. PBM application reversed this process, implying that PMA activated the NF‐ĸB pathway and the expression level of CXCL10 in rats, the activation process could be inhibited by PBM [Figure 8D, *F* (4, 10) = 90.77, *p* < 0.0001, p‐P65/P65; *F* (4, 10) = 142.2, *p* < 0.000, CXCL10; *F* (4, 10) = 32.24, *p* < 0.0001, CXCR3]. In contrast, similar to PBM irradiation, the intraperitoneal injection of the inhibitor PDTC also reduced the activation of the NF‐ĸB pathway and the expression of CXCL10 in SCI rats [Figure 8E, *F* (4, 10) = 50.68, *p* < 0.0001, p‐P65/P65; *F* (4, 10) = 29.67, *p* < 0.0001, CXCL10; *F* (4, 10) = 63.71, *p* < 0.0001, CXCR3]. These results suggest that crosstalk with activation of the NF‐ĸB pathway is involved in the inhibitory effect of PBM on CXCL10 expression in rats with SCI. NP‐related behavior tests were performed accordingly, and the results showed that PBM irradiation alleviated pain behavior in SCI rats, and the NF‐ĸB pathway promoter PMA reversed it. The NF‐ĸB pathway inhibitor PDTC plays a similar role like PBM irradiation on the pain relief for rats with SCI [Figure 9A–C, *F* (6, 35) = 99.51, *p* < 0.0001, mechanical allodynia; *F* (6, 35) = 31.95, *p* < 0.0001, heat hyperalgesia; *F* (6, 35) = 37.12, *p* < 0.0001, cold allodynia].

## 4 DISCUSSION

Our findings support the feasibility of relieving NP after SCI using PBM treatment. SCI results in a local inflammatory response through glial cell activation and release of proinflammatory factors.^82^, ^83^, ^84^, ^85^ The current studies provide evidence that PBM treatment improves the inflammatory response after SCI in adult male rats.^43^, ^86^, ^87^ In this study, we showed that PBM significantly alleviates NP after SCI by downregulating chemokine CXCL10 expression, which is due, at least in part, to the following mechanisms: PBM inhibits the activation of glial cells in the injured tissue, leading to a decrease in NF‐ĸB phosphorylation level, which further suppresses the expression levels of the chemokine CXCL10, and reduces the occurrence of NP.

This study showed significant relief of NP in rats with PBM irradiation treatment, which is similar to previous results.^18^, ^19^, ^20^ However, few studies have investigated the mechanisms of PBM therapy in relieving NP in post‐SCI animals. Janzadeh et al. observed that PBM treatment downregulated the expression levels of the inflammatory factor IL‐6, trophic factors brain‐derived neurotrophic factor, and glial cell‐derived neurotrophic factor, which are closely associated with the occurrence of NP, while promoting the expression levels of glutamic acid decarboxylase 65, a marker of pain‐inhibitory gamma‐aminobutyric acid neurons.^20^ Meanwhile, a study by Hu et al. found that PBM therapy inhibited the occurrence of NP after SCI associated with the regulation of M1/M2 cell balance.^18^ In this study, to investigate the potential mechanism with a comprehensive screening, we screened the molecules that were regulated by PBM intervention after SCI using RNA‐seq and found the top molecule CXCL10 among the pain‐related molecules. It is also showed that interfering with NF‐κB pathway in astrocytes and microglia could regulate CXCL10 production and the emergence of pain behavior in rats. These results showed that downregulation of glial cell chemokine secretion might be a potential mechanism for PBM treatment in NP after SCI. The wavelength of 810 nm used in this study is also distinctive for photobiological treatment of SCI. Although a wavelength of 810 nm is used for PBM irradiation, and has demonstrated a positive role in SCI treatment,^43^, ^87^, ^88^, ^89^, ^90^, ^91^, ^92^ its therapeutic function for NP after SCI has rarely been investigated. In this study, we found PBM with a wavelength of 810 nm is effective at reducing NP after SCI.

PBM treatment has demonstrated a beneficial effect on the prognosis of SCI animals in prior preclinical studies,^93^, ^94^ Reports have shown that PBM irradiation reduces the production of inflammatory factors, modulates the M1/M2 phenotypic polarization balance of immune cells, rescues the death of neurons, and promotes axonal regeneration and functional recovery. However, systematic clinical validation of the PBM intervention for NP in patients with SCI is still lacking. Recently, the clinical translation of PBM treatment in patients with SCI has been further explored. To investigate the safety parameters of PBM in clinical treatment, previous study has been conducted in large animals and patients. Piao et al.^93^, ^94^ measured the transmission efficiency of PBM irradiation in spinal cord tissue using a flexible probe in the spinal canal of dogs. The irradiation intensity of the PBM was measured at nine sites within the 8‐cm‐long spinal canal. Liang et al.^89^ buried photoconductive fibers under the skin of pigs and irradiated at different powers above the injury site in piglets with SCI, and examined the thermal effects and phototoxicity to verify the safety of PBM irradiation. For irradiation in patients, Liang et al.^95^ fixed laser fibers subcutaneously to the spike stick above the patient's injured site, and irradiated patients with previously validated parameters, they found that laser irradiation did not generate a significant increase in the development of complications in SCI patients. The current studies have validated the safety of PBM treatment in clinical practice; however, further research are needed to verify the effectiveness of PBM interventions on NP after SCI, and to verify the optimal parameters for clinical application.

In this study, we found that PBM could have an inhibitory effect on NP after SCI by interfering with the NF‐κB pathway and glial cell polarization, whereas the specific pathways may require further investigation. Since cytochrome *C* oxidase (CCO) has a distinct absorption band at 810 nm (near‐infrared red light region),^96^ the regulation on CCO might be the potential mechanism for 810 nm‐PBM irradiation. CCO, or mitochondrial respiratory chain complex IV, is the terminal enzyme of the mitochondrial electron transport chain, which catalyzes the oxidation of cytochrome *C* and also mediates the reduction of molecular oxygen to water.^97^, ^98^ It is reported that the absorption of photons in CCO will lead to photolysis and release of nitric oxide (NO).^99^ The dissolved NO could effectively neutralize the free radicals induced by hydrogen peroxide (H_2_O_2_). At the same time, NO regulation is an important mechanism to inhibit peroxide production,^100^ NO can form a stable complex between cardiolipin and cytochrome *C*, when the concentration of NO is higher than that required to inhibit lipid peroxidation in the cytochrome *C*‐cardiolipin complex. The NF‐κB pathway is a pathway sensitive to oxidative stress, and the expression of NF‐κB target genes could respond to the action of reactive oxygen species,^101^ PBM might influence the NF‐κB pathway through inhibiting the oxidative stress by facilitating the photolysis of NO. Neurotoxicity of neuroinflammation is regulated by the transcription factor NF‐κB, which is a central regulator of the innate immune inflammatory response in microglia and astrocytes.^102^, ^103^, ^104^ Through the above process, PBM could play a role in the inhibition of NF‐κB pathway and reducing inflammatory polarization of glial cells.

Inflammatory factors contain various cytokines and chemokines, the expression of cytokines have been studied as the potential mechanism for PBM modulation on NP.^18^, ^20^, ^105^ Yet, the role of PBM on chemokines has scarcely been studied. Multiple chemokines including CXCL10, CX3CL1, CXCL13, and CCL2 are expressed in response to tissue inflammation or nerve damage and cause NP.^106^, ^107^, ^108^, ^109^ In this study, the results showed that the pathological trend of chemokine CXCL10 levels was markedly increased after SCI in rats, and PBM administration significantly downregulated the CXCL10 level, accompanied with reduction of NP sensitivity. For the expression of CXCL10 in glia cells, previous studies have observed increased CXCL10 expression level in inflammation‐induced glial cells,^110^, ^111^, ^112^ including astrocytes and microglia in mice infected with hepatitis virus, having autoimmune encephalomyelitis, and in mice with viral encephalomyelitis. In this study, we demonstrate that the expression of CXCL10 is increased in inflammatory‐induced astrocytes and microglia after SCI. In addition, consistent with previous report, CXCL10 was constitutively expressed in neurons.^113^ We demonstrate that release of chemokine CXCL10 from glia cells play an important role on the onset of NP after SCI.

NF‐ĸB is involved in the development of inflammation and NP, and suppression of NF‐ĸB pathway activation is a potential treatment for pain.^114^, ^115^ However, the NF‐ĸB pathway is also involved in numerous physiological processes, and traditional NF‐ĸB pathway inhibitory agents such as nonsteroidal anti‐inflammatory drugs and glucocorticoids which provide excessive inhibition could cause detrimental effects, while administration of receptor inhibitors, enzyme inhibitors or ubiquitinated ligase inhibitors might lead to side effects by affecting additional signaling pathways.^114^ Therefore, therapies that selectively inhibit NF‐ĸB with little effect on other underlying vital activities would have potential benefits for clinical application. In contrast to previous reports,^88^, ^90^, ^116^, ^117^ we found that NF‐ĸB levels in rat spinal cord tissue were downregulated after PBM intervention, accompanied by a decrease in CXCL10 expression level and reduced NP, indicating that PBM may be a promising clinical application for the inhibition of NF‐ĸB pathway in central NP.

The major shortcomings of this study are as follows. First, the study of the PBM‐related signaling pathways lacked investigation and demonstration of the downstream pathway of CXCL10 contributing to NP after SCI. To address this issue, our group will further validate the role of CXCL10/CXCR3 in the development of NP after SCI using CXCR3 receptor inhibitors and CXCL10 protein. Second, we only focused on the effect of PBM on the expression of chemokines in the early phase of SCI. The role of early inhibition of chemokine expression on the chronic pain of SCI remains to be further explored in subsequent experiments. In addition, only adult male SD rats were used, and the suppressive effect of PBM on NP after SCI in female rats and elderly rats still needs to be further assessed. Therefore, we will design appropriate experiments to fully elucidate the mechanisms of PBM to address these shortcomings.

## 5 CONCLUSIONS

Direct irradiation of the SCI area with implanted laser fiber could reduce the expression level of CXCL10 and alleviate NP in rats with SCI by inhibiting CXCL10 secretion in astrocytes and microglia. The potential mechanisms might be complicated, and the inhibition of NF‐κB signal pathway may be involved. Our findings provide new insights into the molecular mechanisms of PBM in NP suppression after SCI.

## AUTHOR CONTRIBUTIONS

Zhihao Zhang, Zhijie Zhu, Xiaoshuang Zuo, Xuankang Wang, Cheng Ju, Zhuowen Liang, and Kun Li conceived the idea of managing neuropathic pain after spinal cord injury using laser irradiation with 810 nm wave length. Jie Zhou and Yangguang Ma designed the study. Liang Luo, Xin Li, Penghui Li, and Zhiwen Song performed the in vitro experiments. Zhijie Zhu, Huilin Quan, and Liang Luo performed the in vivo experiment. Ning Yang, Jie Zhou wrote the manuscript. Zhenzhen Kou, Beiyu Chen, and Tan Ding revised the manuscript. Xueyu Hu and Zhe Wang provided guidance for the project.

## FUNDING INFORMATION

This work was supported by the Natural Science Foundation of China (No. 81070996, No. 81572151), Shaanxi Provincial Key R&D Program (No. 2020ZDLSF02‐05, No. 2021ZDLSF02‐10).

## CONFLICT OF INTEREST STATEMENT

The authors declare that they have no conflict of interest.

## Supporting information


Data S1.

Data S2.

Data S3.

Data S4.

Data S5.

Data S6.

## Data Availability

The datasets used and/or analyzed during the current study are available from the corresponding author on reasonable request.
