# Supplementary material for: Photobiomodulation reduces neuropathic pain after spinal cord injury by downregulating CXCL10 expression
Source: CNS Neurosci Ther. 2023 Jul 20;29(12):3995–4017. doi: 10.1111/cns.14325 (PMC10651991; doi:10.1111/cns.14325)
Supplement: Supplementary file 1 — Data S1. [file CNS-29-3995-s006.docx]

Photobiomodulation therapy laser fibers

The photobiomodulation therapy laser fibers used in this study has been reported in previous studies[1-3]. The laser fiber consists of the front end, main body, and the rear end. The front end of the laser fiber was fixed on the spinous moderately with absorbable sutures and the rear end of the laser fiber was fixed on skin with absorbable sutures. The length of the light emitting part in the main body is 3 cm (Figure S1 A-B). The fiber was wrapped with highly transparent medical silica which could ensure its flexibility and biocompatibility.


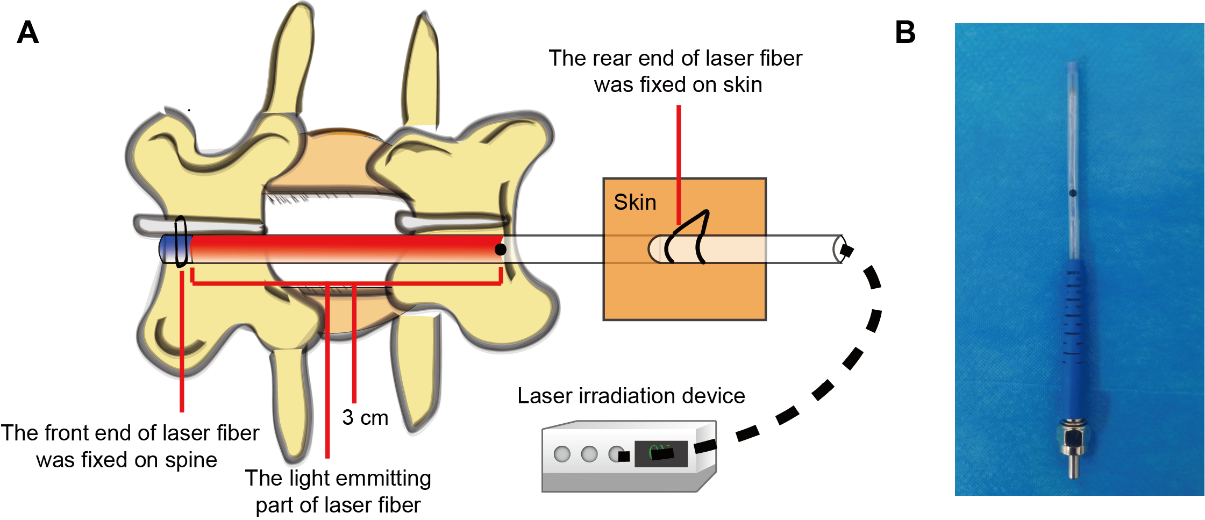


**Figure S1.** Schematic representation of implantation, fixation and irradiation of the laser fiber in rats **(A)**. The physical picture of the laser fiber **(B)**.

References:

1. Liang, Z., et al., *Photobiomodulation by diffusing optical fiber on spinal cord: A feasibility study in piglet model.* Journal of biophotonics, 2020. **13**(4): p. e201960022.

2. Wang, X., et al., *Photobiomodulation inhibits the activation of neurotoxic microglia and astrocytes by inhibiting Lcn2/JAK2-STAT3 crosstalk after spinal cord injury in male rats.* Journal of neuroinflammation, 2021. **18**(1): p. 256.

3. Wang, X., et al., *Photobiomodulation Promotes Repair Following Spinal Cord Injury by Regulating the Transformation of A1/A2 Reactive Astrocytes.* Front Neurosci, 2021. **15**: p. 768262.
