# Supplementary material for: Photobiomodulation reduces neuropathic pain after spinal cord injury by downregulating CXCL10 expression
Source: CNS Neurosci Ther. 2023 Jul 20;29(12):3995–4017. doi: 10.1111/cns.14325 (PMC10651991; doi:10.1111/cns.14325)
Supplement: Supplementary file 2 — Data S2. [file CNS-29-3995-s005.docx]

The protocol of RNA sequencing.

(1) Tissue RNA extraction:

The tissue was ground in liquid nitrogen by adding 1 mL TRIzol per 50–100 mg, and the mixture was homogenized. The samples were incubated at 37 ℃ for 5 min to allow the complete separation of nucleic acid and protein complexes. The supernatant was removed by centrifugation at 12,000 × *g* for 5 min at 4 °C. RNA was present in the supernatant, and the remaining precipitate included cell membranes, polysaccharides, and high-molecular-weight DNA. Chloroform was added at a ratio of 0.2:1 mL TRIzol, shaken intensely for 15 s, and allowed to settle at room temperature for 3 min. The mixture was centrifuged at 4°C for 15 min at 12,000 × *g*. The sample was divided into three layers: a yellow organic bottom, colorless aqueous upper, and intermediate layers. The aqueous phase was transferred to a new Eppendorf tube, and isopropanol was added at a ratio of 0.5:1 mL to precipitate the RNA and place the tube at 4 °C for more than 30 min. The precipitated solution was centrifuged at 12,000 × *g* for 10 min at 4 °C, and the supernatant was removed to obtain RNA. One milliliter of 75% ethanol was added and centrifuged at 8,000 × *g* for 5 min at 4 ℃, and the ethanol was aspirated and sample air dried. Approximately 50–100 μL of RNase-free water was added and the RNA dissolved after a few times of aspiration. A total of 8 M of LiCl solution was added, mixed, and the tube was placed on ice for 1 h. Centrifugation at 13,000 × *g* for 15 min was performed at 4 ℃, and the supernatant was removed to obtain the RNA precipitate. The RNA precipitate was washed with 75% ethanol. A total of 20 μL of RNase-free water was added to dissolve the RNA precipitate.

(2) RNA quality control:

RNA concentration was quantified using 1 μL of RNA solution in a Qubit instrument (Thermo Fisher Scientific, Colorado Springs, CO, USA). Based on the concentration of RNA, 300 ng of RNA was subjected to 1% agarose electrophoresis.

(3) RNA library construction:

A total of 2 μg of total RNA was added to 50 μL of water and 50 μL of mRNA purification beads and mixed well. The sample was placed in a polymerase chain reaction (PCR) instrument, allowed to react at 65 °C for 5 min, and then left at room temperature for 5 min. The sample was placed on a magnetic rack for 5 min, and the supernatant was removed. A total of 200 μL wash buffer was added, mixed well, placed on the magnetic rack again for 5 min, and the supernatant removed. A total of 50 μL of elution buffer was added, mixed well, and the above steps were repeated. A total of 200 μL of binding buffer was added, mixed well, and left at room temperature for 5 min. The mixture was placed on a magnetic rack for 5 min, and the supernatant was removed. A total of 200 μL of wash buffer was added, mixed well, placed on a magnetic rack for 5 min, and the supernatant removed. A total of 19 μL of elute, fragment mix and prime was added, mixed well, and placed in PCR instrument at 94 ℃ for 3 min and at 4 ℃ for 5 min. Sample of 17 μL was removed and added to a new Eppendorf tube, 8 μL of first strand master mix was added to the new tube, and mixed well. A total of 25 μL of second strand master mix was added, mixed well, placed it into a PCR machine, and reacted at 16 ℃ for 1 h. A total of 90 μL of ampure beads (Beckman, Indianapolis, IN, USA) were added, mixed well, and placed at room temperature for 15 min. Then the tube was placed on a magnetic rack for 5 min, and the supernatant removed. This process was repeated two times while adding 200 μL of 80% ethanol, reacted at room temperature for 30 s, and the supernatant discarded. The solution was dried at room temperature for 15 min. A total of 62 μL of resuspension buffer was added, mixed well, and left at room temperature for 5 min. A total of 60 μL of supernatant was added to a new Eppendorf tube.

A total of 40 μL Illumina end repair buffer was added to 60 μL fragmented DNA, mixed well, and centrifuged at 12,000 × *g* for 5 min at 4 °C. A total of 160 μL of ampure beads were added to the solution, mixed well and left at room temperature for 15 min. The solution was placed on a magnetic stand for 5 min, and the supernatant removed. This step was repeated 2 times while adding 200 μL of 80% ethanol, left for 30 s at room temperature, and the supernatant was removed. A total of 20 μL of resuspension buffer was added, mixed, left for 5 min at room temperature. A total of 17.5 μL of supernatant was placed in a new Eppendorf tube.

A total of 12.5 μL of A-tailing buffer was added to 17.5 μL of DNA solution, mixed well, and subjected to PCR for 30 min at 37°C. A total of 2.5 μL of resuspension buffer, 2.5 μL of ligation mix, and 2.5 μL of junction were added to the liquid and mixed well. A total of 5 μL stop ligation buffer was added, mixed well, and 42.5 μL ampure beads were added, and the sample was left at 37 ℃ for 15 min. The mixture was placed on a magnetic rack for 5 min, and the supernatant was removed. This process was repeated two times after adding 200 μL of 80% ethanol and incubating at room temperature for 30 s; the supernatant was removed. Then, 22 μL of resuspension buffer was added, mixed, and incubated for 5 min at room temperature. Twenty microliters of the supernatant were added to a new Eppendorf tube.

Next, 25 μL of PCR mix were added to 20 μL of the DNA solution. The PCR instrument was incubated at 98 ℃ for 30 s, and 10 cycles (98 ℃ for 10 s, 60 ℃ for 30 s, and 72 ℃ for 30 s) were performed. The reaction was performed at 72 °C for 5 min and finally cooled to 10 °C for holding. Ampure beads (50 μL) were then added, mixed thoroughly, and incubated at room temperature for 15 min. The samples were placed on a magnetic stand for 5 min, and the supernatant was removed. The process was repeated two times while adding 200 μL of 80% ethanol, reacted at room temperature for 30 s, and the supernatant removed. After drying at room temperature for 15 min, 32 μL resuspension buffer was added, mixed well, and left at room temperature for 5 min. Then, 30 μL of the supernatant was collected in a new Eppendorf tube. The Qubit quantitative RNA library (Thermo Fisher Scientific) was used for quantification.

(4) Cluster generation and sequence acquisition:

Next, 1 μL of 2 N NaOH and 15 μL of Tris-HCl were added to 4 μL of the solution, mixed, and left at room temperature for 5 min. Then, 6 μL of the liquid was added to 994 μL of cold hybridization buffer. The solution (140 μL) was then placed in an 8-lane tube, further placed in the template column of the cBot, and run in the cBot instrument to complete cluster generation.

NovaSeq 6000 (Illumina, USA) was run, and the sequencing results were obtained. Finally, raw data was converted to FASTQ format.
