# Supplementary material for: Photobiomodulation reduces neuropathic pain after spinal cord injury by downregulating CXCL10 expression
Source: CNS Neurosci Ther. 2023 Jul 20;29(12):3995–4017. doi: 10.1111/cns.14325 (PMC10651991; doi:10.1111/cns.14325)
Supplement: Supplementary file 3 — Data S3. [file CNS-29-3995-s004.docx]

**Table S1. Sequences of primer pairs**

| Genes | Forward primer sequence (5′– 3′) | Reverse primer sequence (5′– 3′) |
| --- | --- | --- |
| CXCL10 | 5′- TGCAAGTCTATCCTGTCCGC-3′ | 5′- CTCTCTGCTGTCCATCGGTC-3′ |
| CXCR3 | 5′- TACCTTGAGGTCAGTGAACGTCA-3′ | 5′- GCTTTCGTTTTCCCCATAATC-3′ |
| IL-18 | 5′-ATGCCTGATATCGACCGAAC-3′ | 5′-TGGCACACGTTTCTGAAAGA-3′ |
| IL-1β | 5′-CAGCAGCATCTCGACAAGAG-3′ | 5′-CATCATCCCACGAGTCACAG-3′ |
| TNF-α | 5′-TACTGAACTTCGGGGTGATCG-3′ | 5′-CCACTTGGTGGTTTGCTACG-3′ |
| IL-6 | 5′-CTTCCATCCAGTTGCCTTCTTG-3′ | 5′-AATTAAGCCTCCGACTTGTGAAG-3′ |
| IL-10 | 5′-CACTCCCTGCATTACAATC-3′ | 5′-CAATGATGGTATTATAGGATCCC-3′ |
| GAPDH | 5′-GAACATCATCCCTGCATCCA-3′ | 5′-CCAGTGAGCTTCCCGTTCA-3′ |
