# Supplementary material for: Photobiomodulation reduces neuropathic pain after spinal cord injury by downregulating CXCL10 expression
Source: CNS Neurosci Ther. 2023 Jul 20;29(12):3995–4017. doi: 10.1111/cns.14325 (PMC10651991; doi:10.1111/cns.14325)
Supplement: Supplementary file 4 — Data S4. [file CNS-29-3995-s002.docx]

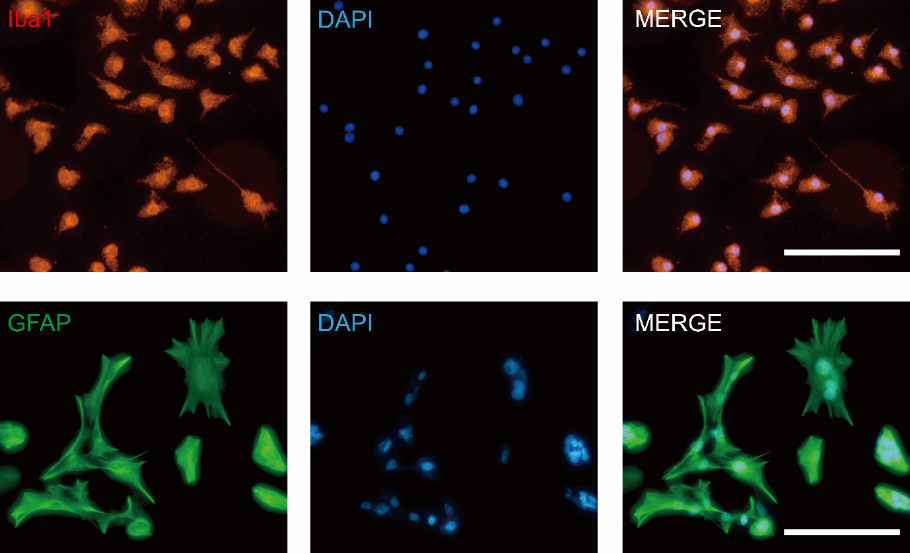


**Figure S2.** Identification of astrocytes and microglia by cellular immunofluorescence staining. Scale bar for all pictures: 200 μm.
