# Supplementary material for: Photobiomodulation reduces neuropathic pain after spinal cord injury by downregulating CXCL10 expression
Source: CNS Neurosci Ther. 2023 Jul 20;29(12):3995–4017. doi: 10.1111/cns.14325 (PMC10651991; doi:10.1111/cns.14325)
Supplement: Supplementary file 5 — Data S5. [file CNS-29-3995-s001.docx]

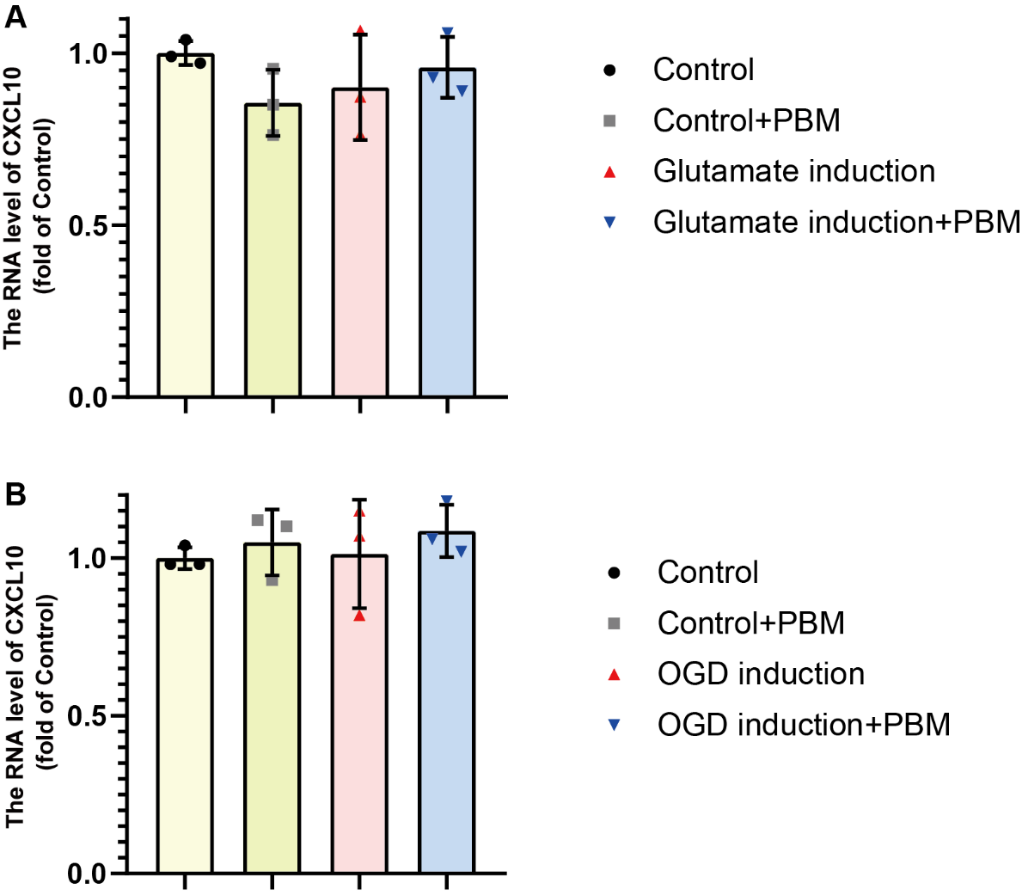


**Figure S3.** Expression levels of CXCL10 mRNA in VSC 4.1 neuron cell line. No significant difference in CXCL10 mRNA expression could be found in cultured neurons after glutamate treatment (7 mM for 8 h) (**A**) or OGD induction (induced for 2h) (**B**) when compared to the control or control+PBM groups. Data is expressed as mean ± SD. OGD: oxygen glucose deprivation; PBM: photobiomodulation; SD: standard deviation
