# Supplementary material for: Photobiomodulation reduces neuropathic pain after spinal cord injury by downregulating CXCL10 expression
Source: CNS Neurosci Ther. 2023 Jul 20;29(12):3995–4017. doi: 10.1111/cns.14325 (PMC10651991; doi:10.1111/cns.14325)
Supplement: Supplementary file 6 — Data S6. [file CNS-29-3995-s003.docx]

**Full unedited blot for Figure 3A**

**
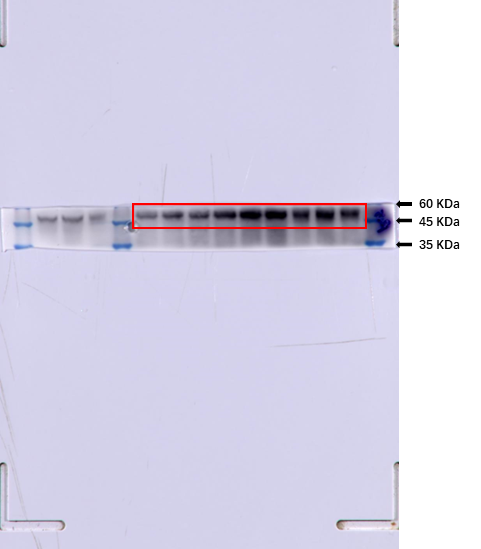
**

**CXCR3**

**
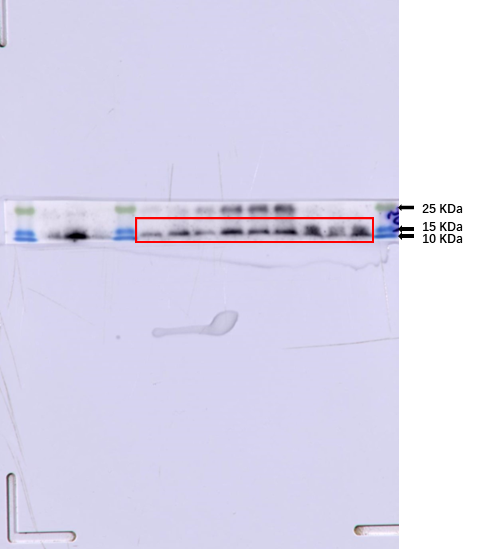
**

**CXCL10**

**
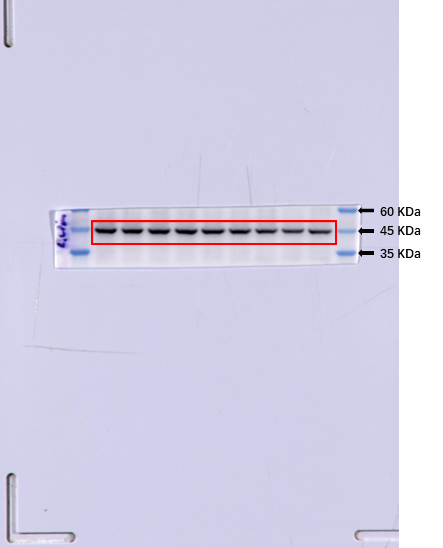
**

**β-actin**

**Full unedited blot for Figure 5C**

**
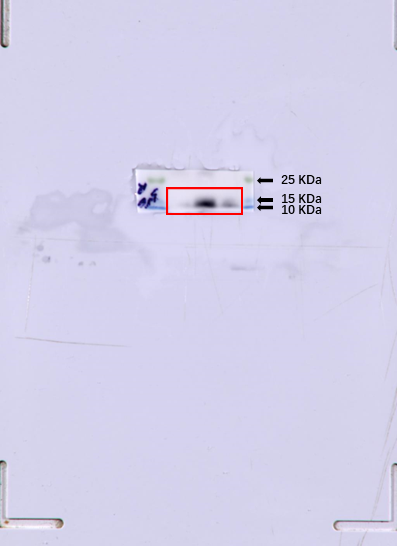
**

**CXCL10**

**
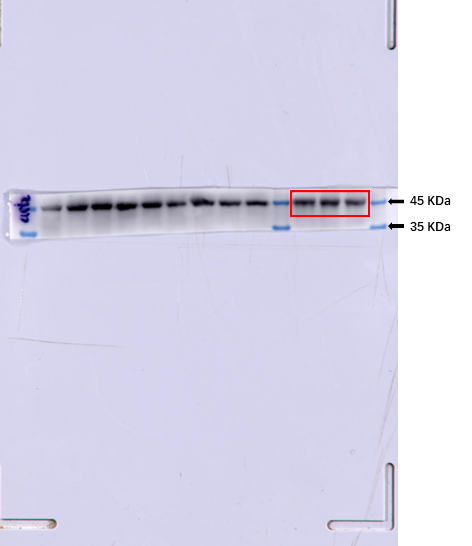
**

**β-actin**

**Full unedited blot for Figure 5F**

**
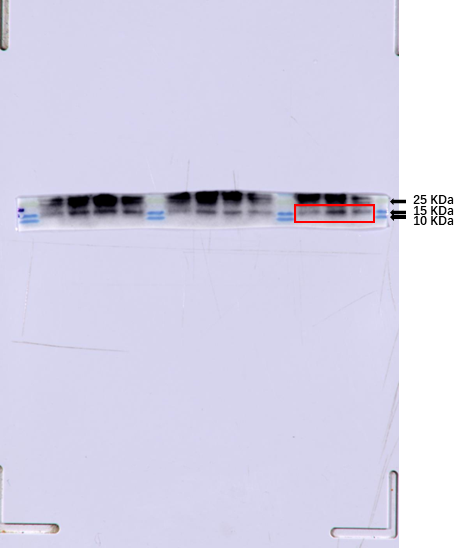
**

**CXCL10**

**
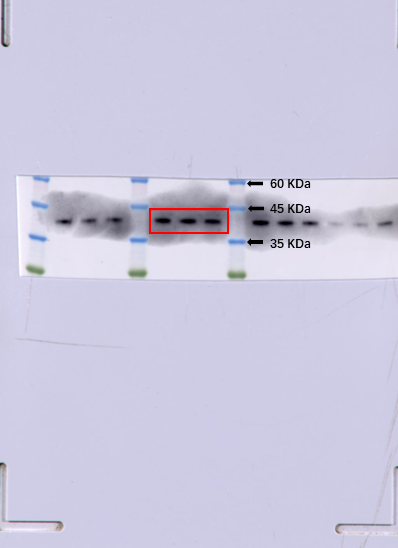
**

**β-actin**

**Full unedited blot for Figure 6B**

**
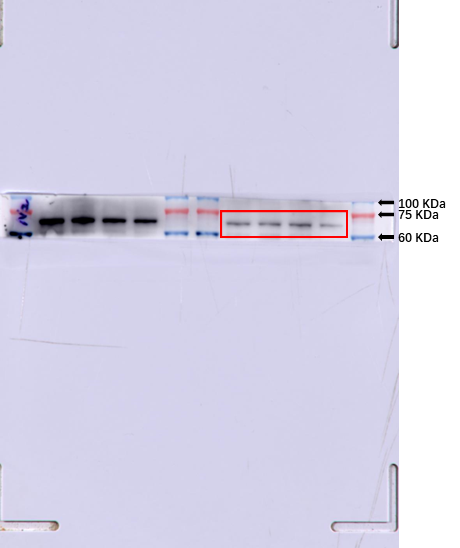
**

**P65**

**
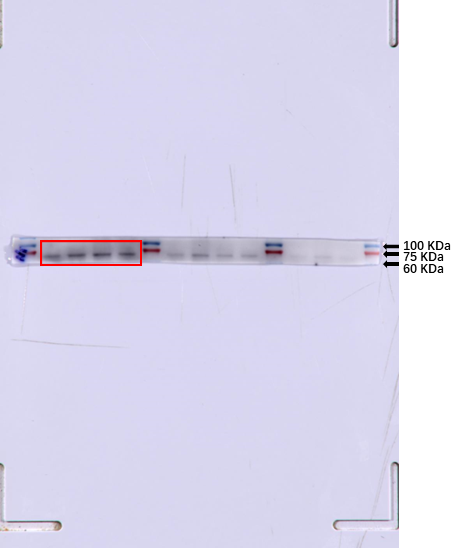
**

**P-P65**

**
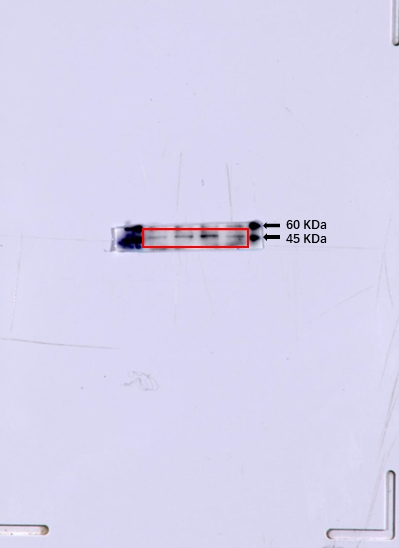
**

**CXCR3**

**
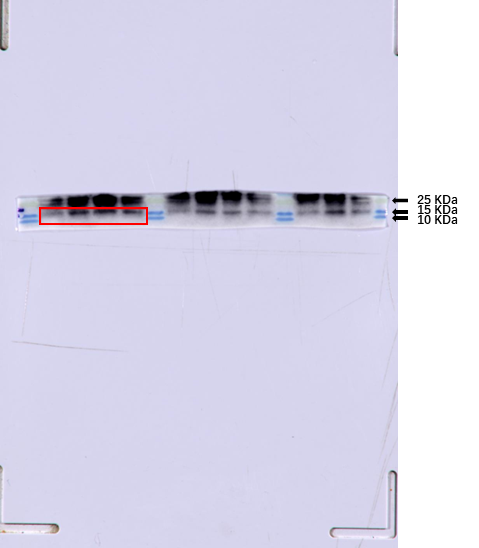
**

**CXCL10**

**
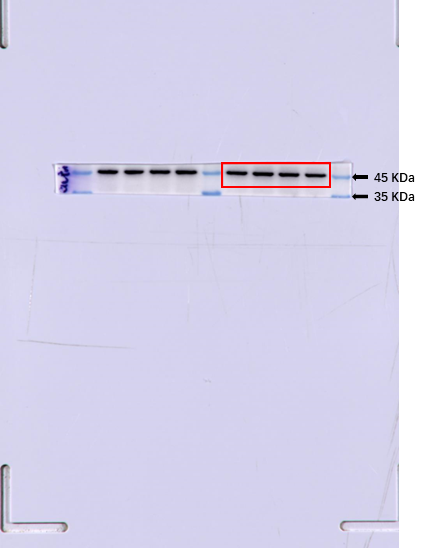
**

**β-actin**

**Full unedited blot for Figure 6C**

**
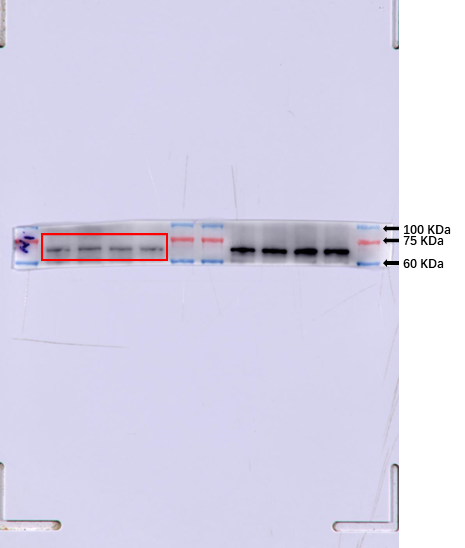
**

**P65**

**
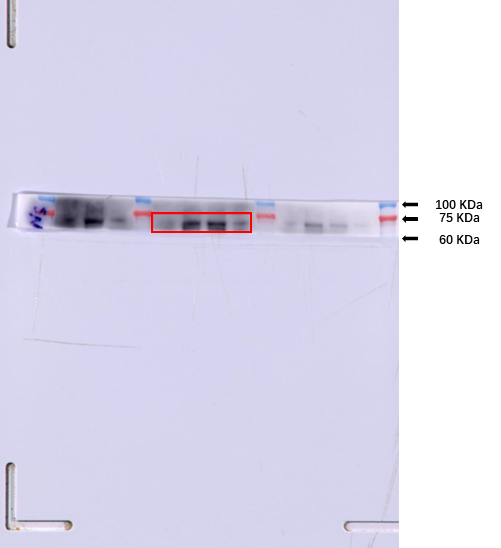
**

**P-P65**

**
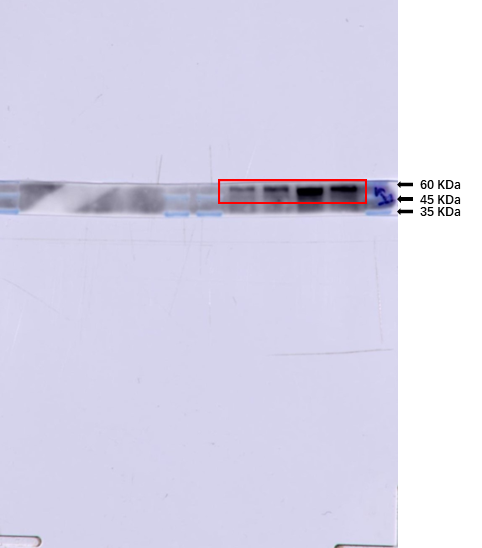
**

**CXCR3**

**
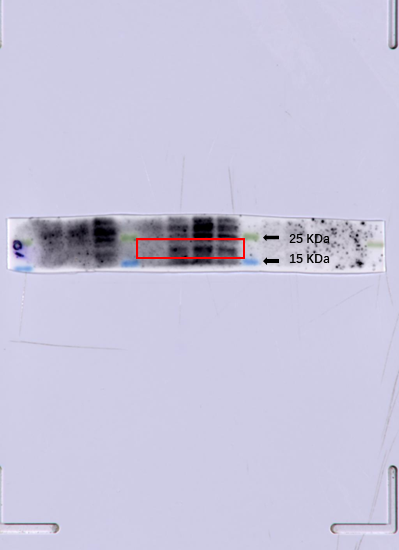
**

**CXCL10**

**
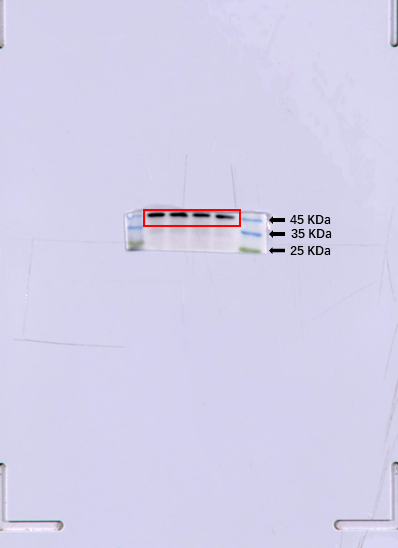
**

**β-actin**

**Full unedited blot for Figure 7B**

**
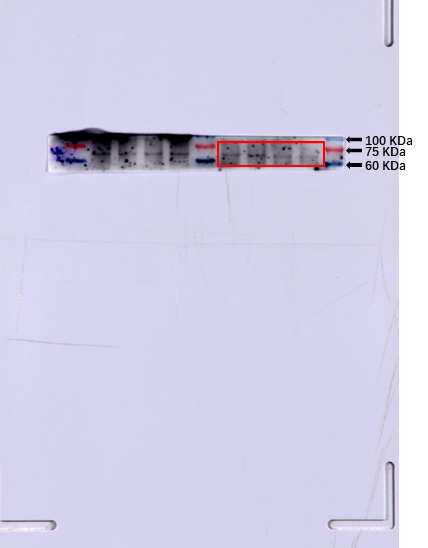
**

**P65**

**
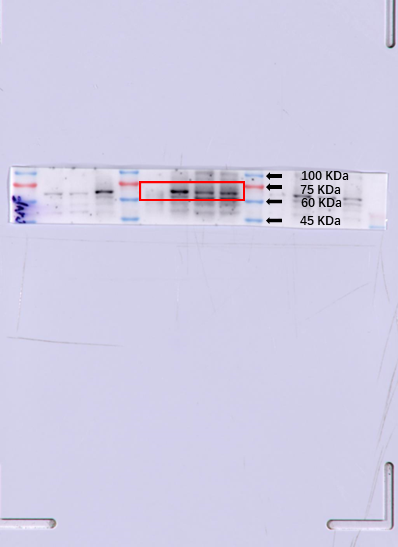
**

**P-P65**

**
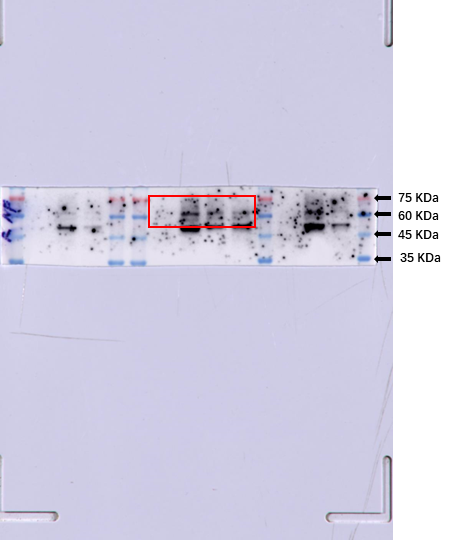
**

**CXCR3**

**
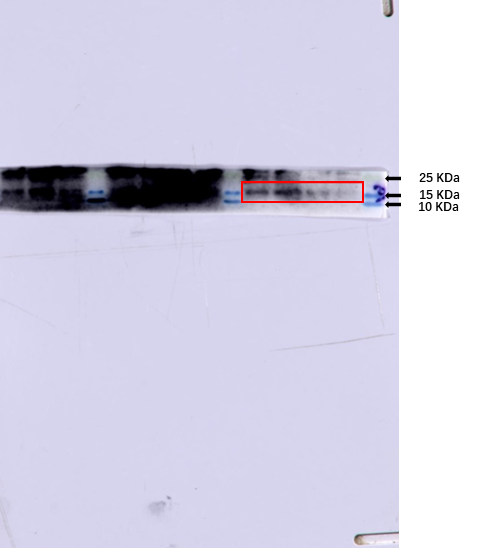
**

**CXCL10**

**
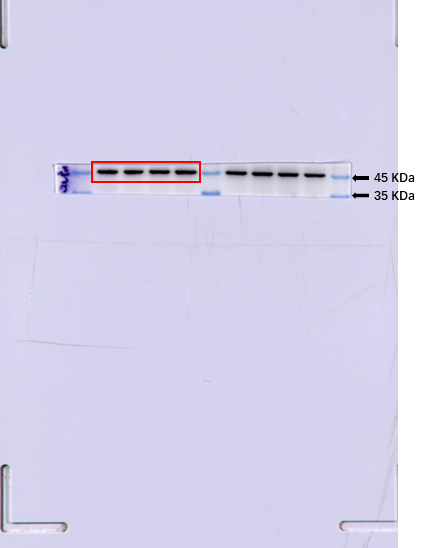
**

**β-actin**

**Full unedited blot for Figure 7C**

**
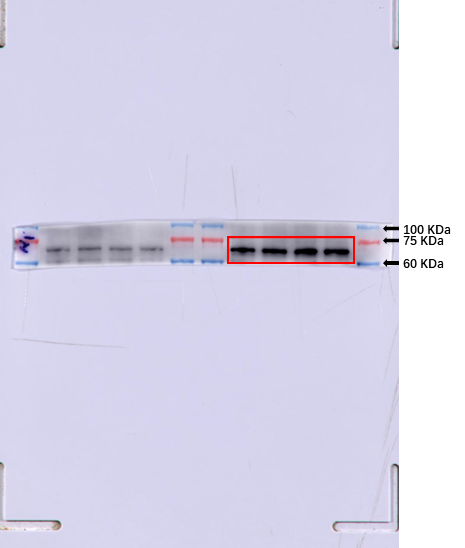
**

**P65**

**
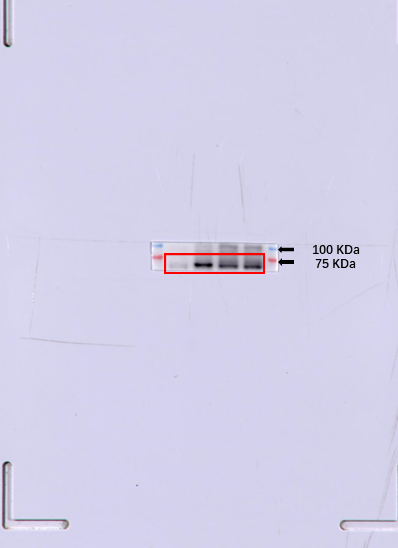
**

**P-P65**

**
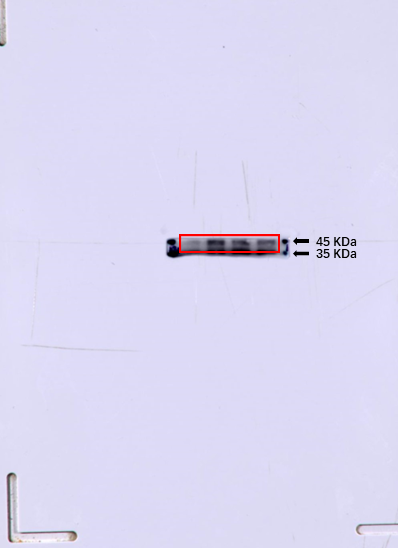
**

**CXCR3**

**
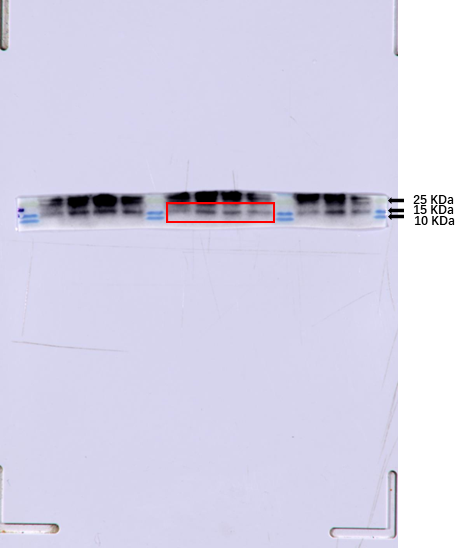
**

**CXCL10**

**
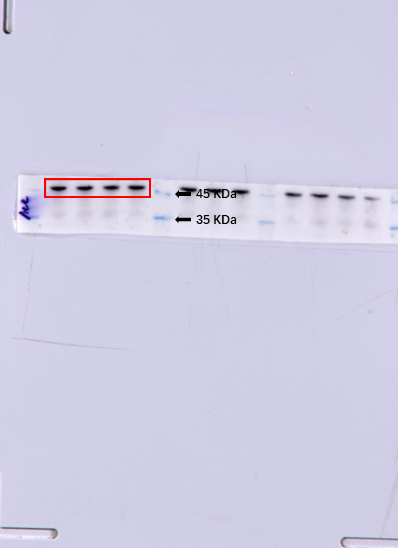
**

**β-actin**

**Full unedited blot for Figure 8D**

**
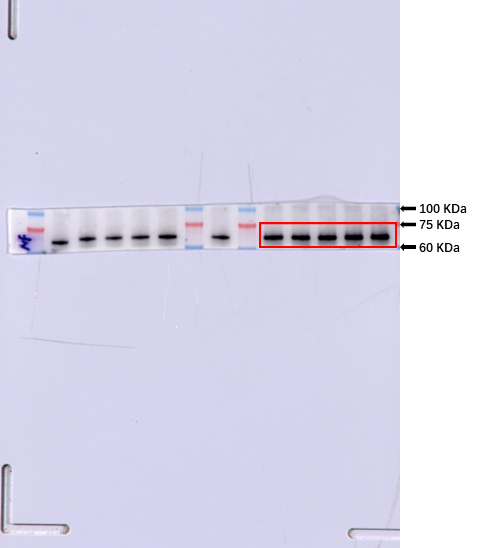
**

**P65**

**
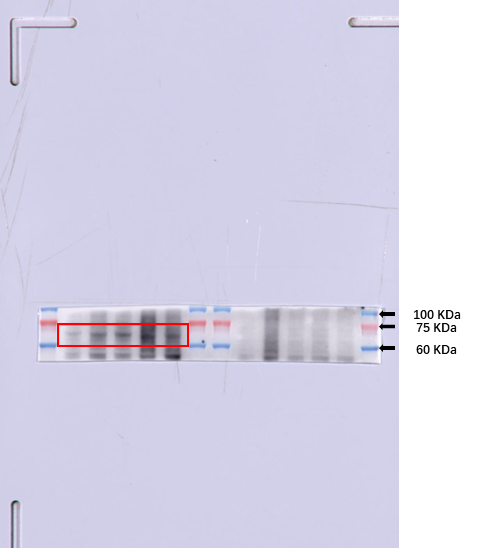
**

**P-P65**

**
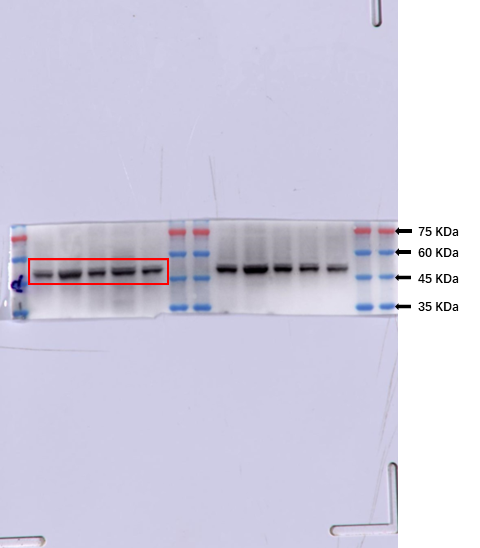
**

**CXCR3**

**
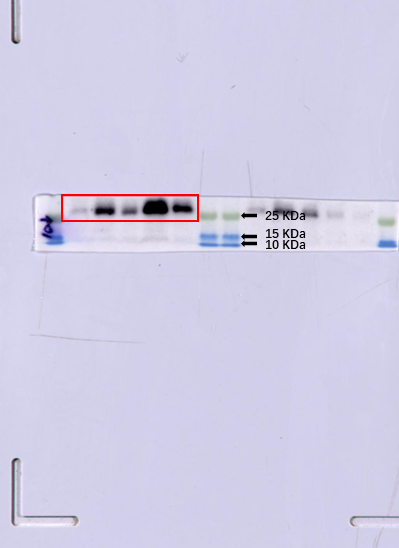
**

**CXCL10**

**
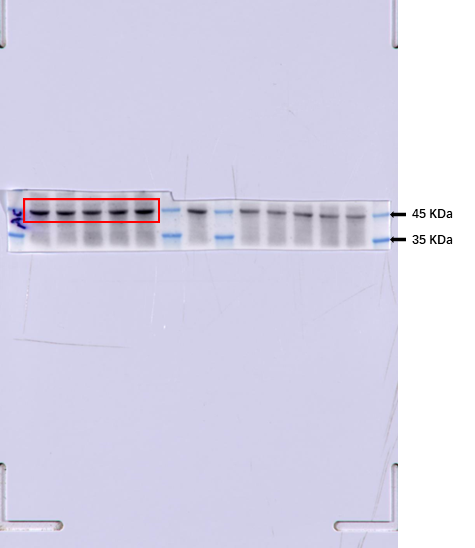
**

**β-actin**

**Full unedited blot for Figure 8E**

**
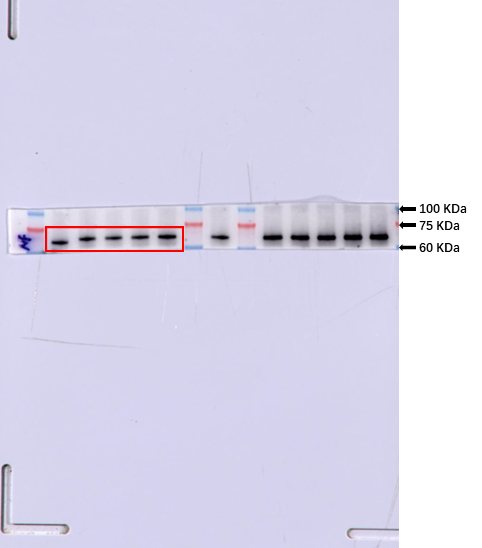
**

**P65**

**
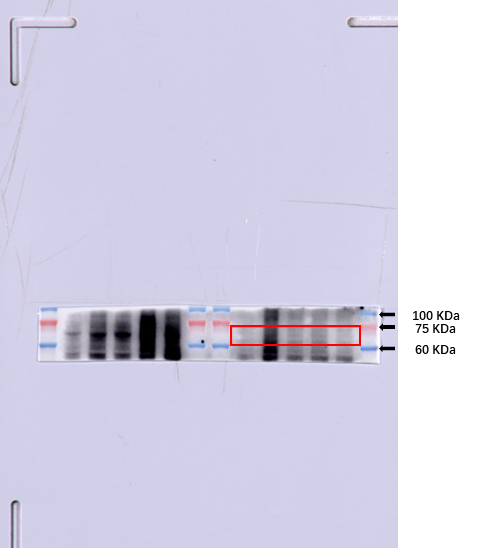
**

**P-P65**

**
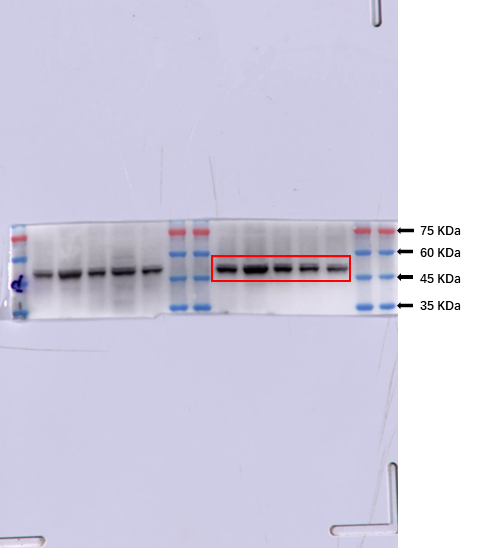
**

**CXCR3**

**
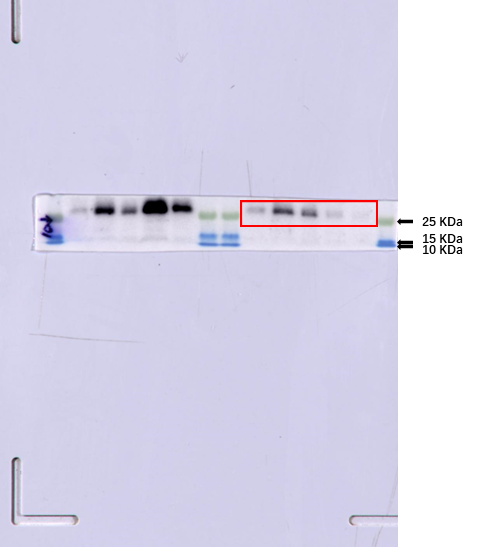
**

**CXCL10**

**
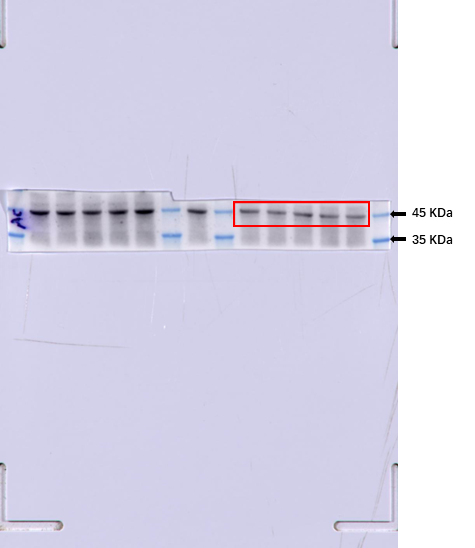
**

**β-actin**
